# *d*-Orbital steered active sites through ligand editing on heterometal imidazole frameworks for rechargeable zinc-air battery

**DOI:** 10.1038/s41467-020-19709-6

**Published:** 2020-11-17

**Authors:** Yi Jiang, Ya-Ping Deng, Ruilin Liang, Jing Fu, Rui Gao, Dan Luo, Zhengyu Bai, Yongfeng Hu, Aiping Yu, Zhongwei Chen

**Affiliations:** 1 Department of Chemical Engineering, Waterloo Institute for Nanotechnology, University of Waterloo, Waterloo, ON N2L 3G1 Canada; 2 School of Materials Science and Engineering, Tongji University Shanghai, 201804 Shanghai, China; 3 School of Chemistry and Chemical Engineering, Henan Normal University, 453007 Xinxiang, China; 4 Canadian Light Source, University of Saskatchewan, Saskatoon, SK S7N 0X4 Canada

**Keywords:** Batteries, Batteries, Batteries

## Abstract

The implementation of pristine metal-organic frameworks as air electrode may spark fresh vitality to rechargeable zinc-air batteries, but successful employment is rare due to the challenges in regulating their electronic states and structural porosity. Here we conquer these issues by incorporating ligand vacancies and hierarchical pores into cobalt-zinc heterometal imidazole frameworks. Systematic characterization and theoretical modeling disclose that the ligand editing eases surmountable energy barrier for *OH deprotonation by its efficacy to steer metal *d*-orbital electron occupancy. As a stride forward, the selected cobalt-zinc heterometallic alliance lifts the energy level of unsaturated *d*-orbitals and optimizes their adsorption/desorption process with oxygenated intermediates. With these merits, cobalt-zinc heterometal imidazole frameworks, as a conceptually unique electrode, empowers zinc-air battery with a discharge-charge voltage gap of 0.8 V and a cyclability of 1250 h at 15 mA cm^–2^, outperforming the noble-metal benchmarks.

## Abstract (web summary)

Low intrinsic activity and accessibility of active sites limit the application of metal-organic framework as catalyst for Zn-air battery. Here, authors present a cation substitution strategy to regulate the electronic state of metal sites and modify its porosity, which enables battery operation.

## Introduction

Rechargeable Zn–air batteries (ZABs) with four-time higher theoretical energy density, better safety, and lower cost are commonly considered as one of the most promising replacements for Li-ion batteries^1–5^. These superiorities mainly originate from oxygen-based electrochemistry in aqueous systems, but their full potential has yet to be realized because of high polarization and short lifespan at the air cathodes^6–8^. As such, the core of ZABs development lies in exploring air cathodes that are capable of efficiently catalyzing both oxygen reduction (ORR) and evolution reactions (OER) for long working periods^9–12^. Platinum on carbon (Pt/C) and ruthenium oxide (RuO_2_) are the respective ORR and OER benchmarks, however, their applications in rechargeable ZABs are not favored due to their scarcity and inferior bifunctionality^7,13^. Previous attempts on economically viable electrocatalysts primarily involve inorganic candidates, such as heteroatom-doped carbon materials, metal compounds or their composites^9,14–24^. However, their electrocatalytic activity and stability are limited by dissolution or aggregation of inorganic components triggered by corrosion in highly concentrated alkaline electrolytes of oxygen free radicals emerged during battery cycling^3,25,26^.

In response, molecules with conjugated heteroaromatic organic components, such as covalent organic frameworks (COFs), hydrogen-bonded organic frameworks (HOFs) and metal-organic frameworks (MOFs), provide a possible solution to effectively resist against the attack from the oxygen free radicals^27–29^. As a subfamily of MOFs, zeolite imidazole frameworks (ZIFs) with excellent water and alkaline tolerance endow great promise in ZABs application^30,31^. Moreover, their ordered coordination manner of single metal nodes and organic ligands provides an ideal platform to regulate the electronic states of active sites and investigate their electrochemical behaviors.

However, application of ZIFs as air electrode in ZABs remains unexplored, which is ascribed to the impediments in controls over mass/charge transfer and intrinsic activity of the materials. Specifically, pristine ZIFs are mostly microporous, which usually leads to severe electrolyte flooding due to capillarity action, and thereby impedes O_2_ accessibility towards active sites^10,27,32^. Besides, their low electrical conductivity inevitably causes sluggish charge transfer^33^. This is often addressed by high-temperature pyrolysis, which causes the dilemma of compromising intrinsic properties, including periodic coordination structure, monodisperse metal nodes, and high surface area^20,34^. Additionally, the pyrolysis imposes side-effects on electrode flexibility due to the inevitable fragmentation of catalysts and degeneration of substrate (e.g., nickel foam, copper mesh, carbon cloth). Lastly, the saturated coordination environment of metal nodes in ZIFs suffers from an adverse electronic structure for oxygen electrocatalysis, as Zn and Co generally coordinate with four N from four dimethyl imidazole to form tetrahedral coordination (*T*_*d*_) in ZIFs^35^. Following the Sabatier principle, these Zn or Co nodes present a completely filled or half-empty *d*-orbitals, which results in bonding with oxygenated intermediates that are either too weak or too strong^36^. Currently, to develop pristine ZIFs for ZABs remains a major challenge, let alone the comprehensive studies on modulating electronic states of metal nodes to profoundly improve battery performance.

In this work, we propose a competing coordination strategy to prepare an air cathode based on vacancy-rich and hierarchically porous Co-Zn heterometallic ZIFs grown on Ni foam. The direct growth of ZIFs on Ni foam and its hierarchical porosity ensure rapid charge and mass transfer. Moreover, this strategy enables the regulation of ligand coordination and generation of unoccupied *3d*-orbitals at metal sites to ease the energy barrier of oxygen electrocatalysis. Meanwhile, increment in *d*-orbital energy level via Co-Zn heterometallic alliance further improves the intrinsic activity of active sites, as evidenced by the optimal electrocatalytic performance of BHZ-48.

## Results

### Physicochemical characterizations

**Fig. 1:**
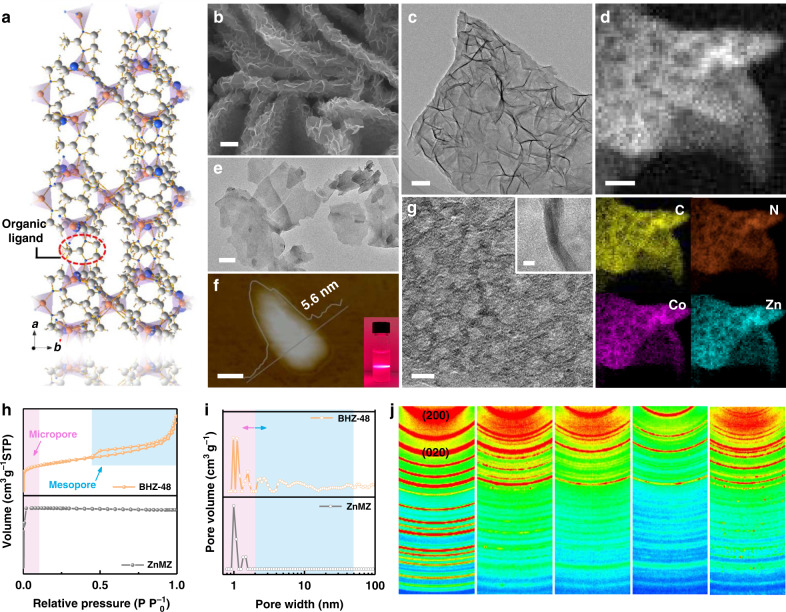
Morphology and structural properties. **a** Schematic of the crystal structure of ZnMZ. **b** SEM and **c** TEM images of BHZ-48. **d** Scanning TEM images and corresponding EELS elemental maps of C, N, Co, and Zn atoms. **e** TEM, **f** AFM images and corresponding height curves of ultrasonically detached fragment from BHZ-48. Inset: Tyndall light scattering of BHZ-48 nanosheets dispersed in aqueous solution. **g** High-resolution TEM image of BHZ-48. **h** N_2_ adsorption–desorption isotherms, **i** pore-size distribution of ZnMZ and BHZ-48. **j** Two-dimensional WAXS patterns of BHZ-48 and comparison samples, red color represents region with higher diffraction intensity. Scale bars: 1 μm (**b**), 200 nm (**c**), 10 nm (**d**), 100 nm (**e**), 50 nm (**f**), 5 nm (**g** and inset of (**g**)).

As the precursor, pristine Zn-based ZIFs arrays with average lateral length of three micrometers were grown on Ni foam (referred as ZnMZ) through a low-temperature solvothermal reaction (Supplementary Fig. 1a, e and 2). The crystal framework of ZnMZ is assembled by Zn^II^ ions and dimethyl imidazole (DI) in the form of [ZnN_4_] tetrahedral-like geometry, where Zn^II^ ions are fully coordinated with the DI ligands via four N atoms (Fig. 1a)^20^. These quasi tetrahedra are then further edge/corner connected with each other to afford a two-dimensional structure^20^.

To synthesize heterometallic ZIFs with hierarchical pores and ligand vacancies (*V*_L_), the pristine ZnMZ arrays were subjected to a cation-substitution treatment with Co^II^ source for varying time lengths. A series of samples were obtained and named as BHZ-12, BHZ-24, BHZ-48, and BHZ-96, in which the numbers denote the substitution time in hours (Supplementary Fig. 1b–d, f–h). Scanning electron microscopy (SEM) and transmission electron microscopy (TEM) images unveil the morphology transformation from smooth arrays in ZnMZ to rough microsheets with a surface layer of partially exfoliated nanosheets (Fig. 1b, c and Supplementary Fig. 1). The high-angle annular dark-field scanning transmission electron microscopy image and corresponding electron energy loss spectroscopic (HAAD-EELS) elemental mappings show homogeneous elemental distribution throughout the nanosheets (Fig. 1d). Moreover, the ultrasonically broken fragment of BHZ-48 shows low imaging contrast and Tyndall light scattering, which confirm their ultrathin nature and colloidal dispersibility (Fig. 1e, f)^37^. According to the atomic force microscopy (AFM) image (Fig. 1f), the thickness of the nanosheet in BHZ-48 is 5.6 nm. This is further proven in their cross-sectional high-resolution TEM (HRTEM) image shown in inset of Fig. 1g. Unfortunately, due to rapid degradation of ZIFs under electron irradiation, visualization of their lattice fringes by HRTEM was not successful^38^. Interestingly, the emergence of uniform mesopores with width of ~5 nm is detected in BHZ-48 (Fig. 1g), but is absent in ZnMZ (Supplementary Fig. 3). The coexistence of mesopores and intrinsic micropores in BHZ-48 is further confirmed by N_2_ isotherms with a type-IV hysteresis loops (Fig. 1h, i and Supplementary Table 1). According to previous reports, micropores with potential to host active sites contribute most of the catalytic activity, and mesopores are required to transport the reactant and product efficiently toward and away from the catalytic sites in the micropores^39–41^. Thus, such hierarchical pore structures of BHZ-48 are favorable for propelling the oxygen electrocatalysis. Similarly, both BHZ-24 and BHZ-96 manifest hierarchical porosity (Supplementary Fig. 4). In contrast, the absence of hysteresis loop in ZnMZ isotherms clearly demonstrates its micropore dominant nature. It should be pointed out that such hierarchical porosity cannot be achieved via one-pot synthesis of Co-Zn bimetallic ZIFs (denoted as BMZ) using the same stoichiometric ratio of Zn(NO_3_)_2_, Co(NO_3_)_2_ and DI, as smooth and thick nanosheets are generated instead (Supplementary Fig. 5). The key to the successful introduction of mesopores lies in the selected substitution process. During which, the acidic environment created by Co^II^ hydrolysis gradually cleaves the Zn–N coordination bonds, allowing the Co^II^ to coordinate with the released DI and join into the ZnMZ framework^42,43^. The Co^II^ presents half-empty *d*-orbital occupation, which gives rise to much stronger coordination capability than Zn^II^. As a consequence, equilibria is perturbed by competing DI coordination between Co and Zn sites, leading to mismatched crystal growth and the formation of defect-induced mesopore^44^.

**Fig. 2:**
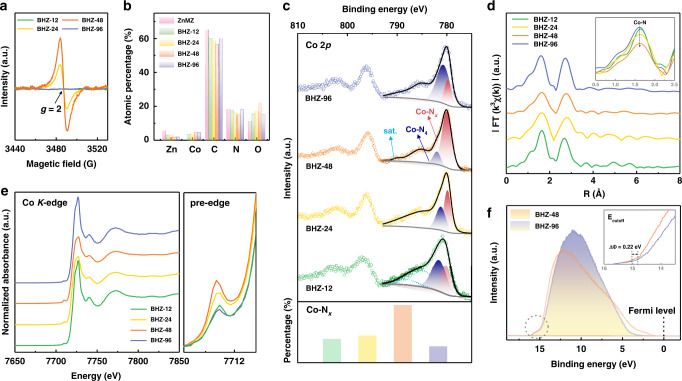
Chemical composition, electronic state, and coordination environment. **a** Electron-paramagnetic resonance spectra, **b** XPS derived element contents, **c** high-resolution Co 2*p* XPS spectra, **d** R-space Co *K*-edge EXAFS spectra, and **e** Co *K*-edge XANES spectra as well as the amplified pre-edges of samples in the BHZ series. **f** UPS spectra of BHZ-48 and BHZ-96 collected using an He I (21.2 eV) radiation. Inset: enlarged view of the secondary electron tail threshold.

Wide-angle X-ray scattering (WAXS) technique is carried out to investigate the crystallographic features of the materials. As shown in Fig. 1j and Supplementary Fig. 6a, these samples exhibit similar crystal structure with ZnMZ, which is in good agreement with the lamellar ZIFs^20^. Comparatively, as the cation-substitution time increases, gradual lattice expansion is reflected by the left shift of diffraction peaks in BHZ-12, BHZ-24, and BHZ-48 (Supplementary Fig. 6b), which is consistent with the powder X-ray diffraction result (Supplementary Fig. 7). Moreover, the significant decrease in diffraction intensity of BHZ-48 reveals reduction in long-range order after the cation-substitution treatment, which originates from defect generation in the above-mentioned competing coordination process (Fig. 1j and Supplementary Fig. 8)^45^. Specifically, the Zn and Co ions dynamically compete for the ligand sites, thus re-coordination of Co and cleavage of Zn coordination bond can both occur during the process. As such, defect (*V*_L_) can be generated on both Co and Zn sites within BHZ that are in the intermediate equilibrium states. The presence of defects are validated by spikes of the peak at *g* = 2 in electron-paramagnetic resonance (EPR) spectra that is commonly associated with unpaired electrons (Fig. 2a)^17^.

The *V*_L_ influence on the chemical composition and coordination condition of ZIFs is investigated by X-ray photoelectron spectroscopy (XPS) and X-ray absorption spectroscopy (XAS). The simultaneous decreases of C and N contents suggest the gradual release of DI ligands in BHZ-12, BHZ-24, and BHZ-48 (Fig. 2b and Supplementary Fig. 9). As for BHZ-96, its C and N content recovers to a similar level as pristine ZnMZ, which are in good agreement with the elemental analysis (EA) results (Supplementary Table 2). The variations in the number of N coordinated to central Co sites are supported by fitted Co 2*p*_3/2_ XPS spectra (Fig. 2c). The peak at 781.1 eV is attributed to fully coordinated Co in a Co-N_4_ environment, and the peak at 779.9 eV is assigned to unsaturated cobalt species in Co-N_*x*_ (*x* < 4) geometry. The Co-N_*x*_ to Co-N_4_ peak intensity ratio experiences a clear increase from BHZ-12 to BHZ-48, indicating reduced Co-N coordination number. Then, cobalt species in unsaturated geometry (Co-N_*x*_) decreases from 57.1% in BHZ-48 to 16.6% in BHZ-96. Considering their similar Co contents by inductively coupled plasma atomic emission spectroscopy (ICP-AES), the composition difference is ascribed to re-coordination of DI ligands at unsaturated orbitals of Co sites (Supplementary Table 2). High-resolution Co *2p* XPS spectra of samples obtained in a substitution treatment with Co^II^ source for 60, 72, and 84 h (denoted as BHZ-60, BHZ-72, and BHZ-84) further reveal the re-coordination phenomena (Supplementary Fig. 10). The vanished EPR signal of BHZ-96 also validates defect removal due to re-coordination and reversion from metal-N_x_ to saturated metal-N_4_ geometry (Fig. 2a). In order to verify the transformation, k-space and Fourier transform (FT) R-space of extended X-ray absorption fine structure (EXAFS) spectra are further analyzed to investigate the Co coordination condition. As demonstrated in Fig. 2d and Supplementary Fig. 11, despite having similar type of Co-N and Co-C coordination shells, BHZ-48 presents the lowest Co-N peak intensity, implying minimal Co-N coordination number. Fitting result of Co-N peak shows an average bond length of ~2.10 Å, while the corresponding coordination number is 2.9 for BHZ-48 and 4.3 for BHZ-96 (Supplementary Table 3). In Co *K*-edge k^3^χ(k) spectra (Supplementary Fig. 12), BHZ-48 exhibits lower oscillation amplitude than BHZ-96, suggesting more disordered local atomic arrangements in the presence of *V*_L_, in consistent with the decrease in WAXS intensity observed on BHZ-48^46^. Similar results are also obtained in Zn *K*-edge EXAFS spectra and corresponding FT curves (Supplementary Fig. 13). The coordination environment of Co center with *T*_*d*_ symmetry is further discerned by the 1*s* → 3*d* transition pre-edge shoulder at ~7709 eV in Co *K*-edge X-ray absorption near edge structure (XANES, Fig. 2e)^17^. The overall XANES spectral shapes of the four samples are similar, with subtle changes observed in pre-edge positions. Given that the pre-edge structure is an indicator of *d*-orbital occupancy^47^, the highest pre-edge peak of BHZ-48 confirms its lowest *d*-orbital occupancy along with increased *V*_L_ abundancy (inset of Fig. 2e). The difference in *d*-orbital occupancy may contribute to distinct work function (Φ) with altered band positions relative to vacuum. This is clearly reflected by ultraviolet photoemission spectroscopy (UPS) spectra, in which the smaller Φ of BHZ-48 (6.18 eV) compare to BHZ-96 (6.40 eV) suggests lower energetic barrier for transferring electrons between active sites and adsorbed intermediates (Fig. 2f)^48,49^. Base on the above results, the *V*_L_ formation and decreased occupancy of Co *d*-orbital states are evidenced to promote electron injection/extraction in O^2−^/OH^−^ redox and hence accelerates the reaction kinetics.

### Electrochemical behaviors

**Fig. 3:**
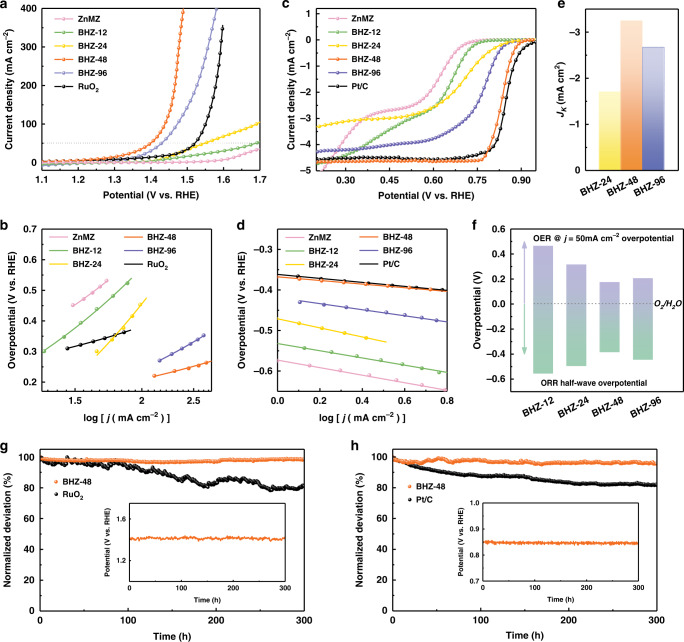
Electrochemical behaviors in half-cell. **a** OER LSV curves, **b** OER Tafel plots comparison, **c** ORR LSV curves, **d** ORR Tafel plots comparison of samples in the BHZ series, commercial RuO_2_, and Pt/C. **e** Comparison of *J*_*K*_ @ half-wave potential, **f** overpotential differences between the *E*_half-wave_ of ORR and *E*_*j* = 50_ of OER for BHZ-48 and other products. **g** OER chronoamperometric response at a fixed overpotential of 170 mV for BHZ-48 and 290 mV for RuO_2_, **h** ORR chronoamperometric response of BHZ-48 and Pt/C at a fixed potential of 0.7 V vs. RHE. Inset: chronopotentiometric response at a constant anodic and cathodic current density for BHZ-48.

Subsequently, the oxygen electrocatalytic activities of the synthesized ZIFs as working electrodes are evaluated in a three-electrode system with 0.1 M KOH as electrolyte. Commercial RuO_2_ powder loaded on Ni foam was prepared as the OER performance benchmark. As illustrated in Fig. 3a, BHZ-48 displays high activity, achieving the lowest overpotential (170 mV) at the current density of 50 mA cm^−2^ (*E*_*j* = 50_) among all samples. It is worth to mention that minimal anodic current density is measured from bare Ni foam when compared to the catalyst-grown counterparts (Supplementary Fig. 14), which suggest that the high OER activity is solely contributed by the ZIFs catalysts. The relationship between *V*_L_ and OER activity is also revealed in the comparison between BHZ-48 and BHZ-96. At a low overpotential of 260 mV, BHZ-48 achieves a current density of 400 mA cm^−2^, which is approximately three times higher than the 125 mA cm^−2^ of BHZ-96, suggesting correlation between unsaturated coordination and improved activity. The smallest Tafel slope of BHZ-48 (80 mV dec^−1^) also indicates the highest reaction kinetics (Fig. 3b).

As for ORR performance, linear sweep voltammetry (LSV) is employed to evaluate the as-synthesized ZIFs with commercial Pt/C as performance benchmark (Fig. 3c). BHZ-48 surpasses others with onset potential (*E*_onset_) of 0.90 V, half-wave potential (*E*_half-wave_) of 0.84 V, as well as limiting current of –4.60 mA cm^–2^ (Supplementary Table 4). To further confirm the electrocatalytic kinetics of BHZ-48, LSV curves at different rotating speeds were collected and corresponding Koutecky–Levich (K–L) plots were analyzed (Supplementary Fig. 15), which indicates an apparent four-electron reduction from oxygen to OH^−^ in BHZ-48. Among catalysts with analogous four-electron reduction, BHZ-48 exhibits the smallest Tafel slope and highest kinetic current density (*J*_*K*_), which further implies its most favorable ORR kinetics (Fig. 3d, e and Supplementary Table 5).

Such bifunctionality arises from the pore reformation and accelerated reactants transport. This is proven by larger electrochemically active surface area (ECSA) of BHZ-48, derived from the calculated electrochemical double-layer capacitance (*C*_dl_ = 25.96 mF cm^−2^), which is 10 times higher than pristine ZnMZ of 2.50 mF cm^−2^ (Supplementary Fig. 16). The catalytic activities of these ZIFs are further normalized by their ECSA, to isolate and identify the enhancement in intrinsic activity from the unsaturated Co *d*-orbitals (Supplementary Fig. 17). Then, the differences (Δ*E* = *E*_*j* = 50_ − *E*_half-wave_) between OER potentials at 50 mA cm^–2^ (*E*_*j* = 50_) and ORR half-wave potentials (*E*_half-wave_) are calculated to directly elucidate the bifunctional activity as compared in Fig. 3f. As shown, BHZ-48 exhibits the smallest Δ*E* of 0.56 V, which outperforms all counterparts and also well-developed electrocatalysts in literatures^9,11,42,50,51^.

Additionally, electrochemical analyses of various protocols are carried out to comprehensively evaluate the stability of BHZ-48. In Fig. 3g, BHZ-48 demonstrates superior OER stability compared to RuO_2_ with only 3% activity loss after 300 h of continuous chronoamperometry tests, which is consistent with the steady activity trend in chronopotentiometry test. It was also found that BHZ-48 retains its OER activity with negligible decrease in *E*_*j* = 50_ (171 mV) after 3000 cycles of cyclic voltammetry (CV) scans, while RuO_2_ catalyst experience an increase from 290 to 300 mV (Supplementary Fig. 18a). As for the ORR performance, despite having activity slightly inferior to Pt/C, BHZ-48 achieves a near 100% retention that is superior to 80% of Pt/C (Fig. 3h). Three-hundred hours of continuous electrocatalysis at constant current also brings negligible decay in potential (inset of Fig. 3h). The durability of BHZ-48 is further confirmed in CV tests without detectable activity loss after 3000 CV cycles (Supplementary Fig. 18b), which should be ascribed to its stable structure and morphology as evidenced by post-cycling characterizations in Supplementary Figs. 19 and 20.

**Fig. 4:**
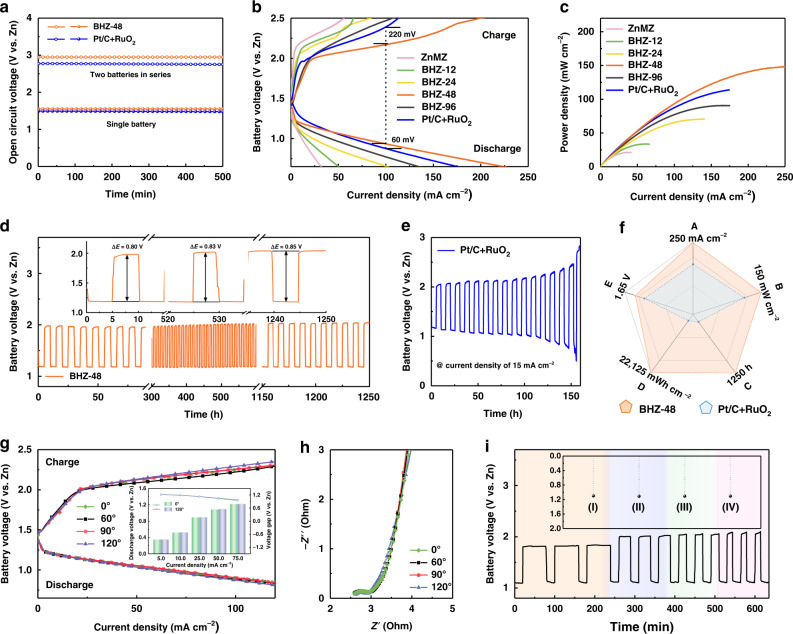
Zn–air battery performance evaluation. **a** Open-circuit voltage curves, **b** charge and discharge polarization curves, and **c** corresponding power density plots for Zn–air battery prepared with BHZ-48 or Pt/C + RuO_2_ electrodes. **d** Galvanostatic cycling stability of Zn–air battery prepared with BHZ-48 electrode, and **e** the Pt/C + RuO_2_ electrode at a current density of 15 mA cm^−2^. **f** Comparison of key performance parameters for BHZ-48 and Pt/C + RuO_2_ electrodes. A: current density; B: power density; C: lifespan, D: cumulative energy density; E: discharge voltage. **g** Charge and discharge polarization curves, inset: corresponding discharge voltages and voltage gaps. **h** Nyquist plots of the flexible Zn–air battery prepared using the BHZ-48 electrode under different bending angles. **i** Alternating charging mode test, (I) 10 mA cm^−2^, 60 min, (II) 20 mA cm^−2^, 30 min, (III) 30 mA cm^−2^, 20 min, (IV) 40 mA cm^−2^, 15 min. Inset in **i**: average discharging voltage variation.

Next, BHZ-48 is directly applied as the air cathode in ZABs. The open-circuit voltage of BHZ-48 is 1.49 V (vs. Zn) and 2.95 V in series, which are higher than the values of commercial Pt/C + RuO_2_ reference (1.44 V and 2.77 V, Fig. 4a). This result aligns well with the lower internal resistance of BHZ-48 (1.6 Ω) measured in electrochemical impedance spectroscopy (EIS, Supplementary Fig. 21a). Figure 4b compares the charge and discharge polarization curves of different electrodes. Among them, BHZ-48 exhibits the narrowest voltage-gap and highest power density of 148 mW cm^–2^ at 250 mA cm^–2^. This maximal power density also outperforms 113 mW cm^–2^ of Pt/C + RuO_2_ (Fig. 4c). The cyclability in ZABs was tested by galvanostatic discharge-charge method at a current density of 15 mA cm^–2^. As shown in Fig. 4d, ZABs with BHZ-48 delivers an initial potential of 1.18 V for discharge and 1.98 V for charge, respectively. After 1250 h of operation, the voltage-gap increases by only 0.05 V, which converts to an low fading rate of 0.004% per hour (Supplementary Fig. 21b). As for the Pt/C + RuO_2_ reference, it shows rapid voltage-gap expansion from 0.87 V to 1.63 V after 150 h with a fading rate of 0.51% per hour, which is two orders of magnitude faster than BHZ-48 (Fig. 4e). The robust corrosion resistance of BHZ-48 is further evident by its obviously lighter electrolyte color than commercial Pt/C + RuO_2_ after cycling (Supplementary Fig. 21c). Overall, this air electrode outperforms commercial noble metals in key aspects (Fig. 4f), and is also comparable to or even better than most reported ZABs to date (Supplementary Table 6).

To explore the feasibility of BHZ-48 in practical application, a flexible ZAB is assembled as shown in Supplementary Fig. 22. Figure 4g and Supplementary Fig. 23 present its output voltage and power density as a function of current density under various bending angles. The voltage polarization of this battery remains virtually unchanged at a high current density of 120 mA cm^–2^ for both charge and discharge. Particularly, negligible increase is detected at 120° and a maximum power density of 100 mW cm^–2^ is achieved. Additionally, EIS results of the battery assembled with BHZ-48 are presented in Fig. 4h. The closely overlapping semicircle and tail in Nyquist plots suggest similar charge transfer and interfacial resistances in different bending conditions. Another desired property for rechargeable batteries is fast-charging capability, but it remains a huge challenge to achieve without negative influence on battery performance^52,53^. Given so, flexible ZAB with BHZ-48 electrode was tested under alternating charging mode (slow, even, fast). As depicted in Fig. 4i, both of its discharging voltage and the capacity are maintained, and the remarkable reversibility is showcased. These features demonstrate the robust physical stability, decent rechargeability, and conductivity of the BHZ-48 electrode, conferring its great promise in practical application.

### Mechanistic study

**Fig. 5:**
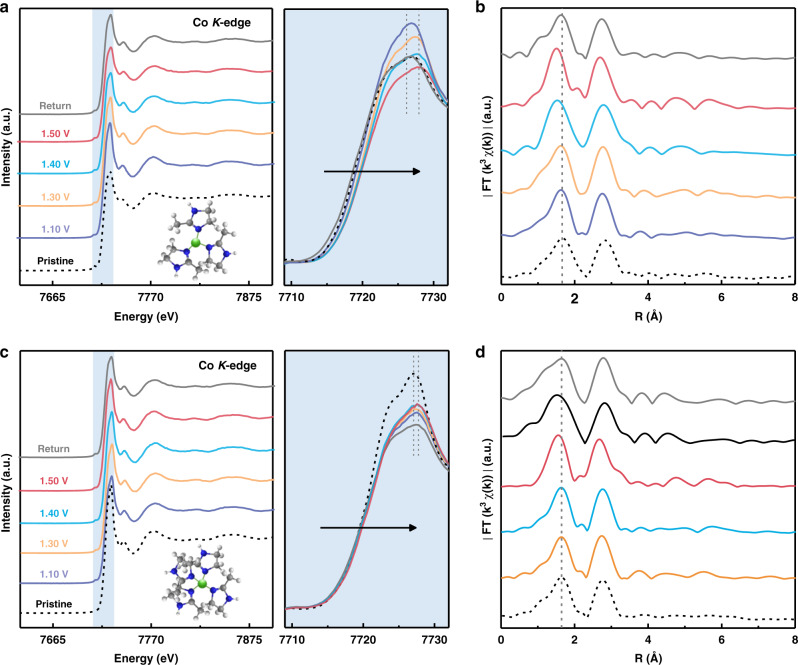
X-ray absorption spectroscopic analysis. Co *K*-edge (**a**) XANES spectra and (**b**) EXAFS in R-space of as-prepared BHZ-48 collected during potentiostatic OER experiments at various potentials. Co *K*-edge (**c**) XANES spectra and (**d**) EXAFS in R-space of as-prepared BHZ-96 collected during potentiostatic OER experiments at various potentials.

**Fig. 6:**
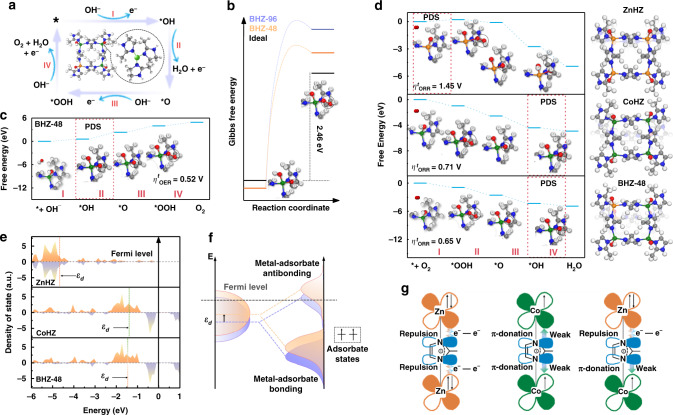
DFT simulations and proposed mechanism illustration. **a** Proposed OER catalytic mechanisms for BHZ-48. **b** Calculated energy barriers of the *OH transformation to *OOH at active sites of BHZ-48 and BHZ-96. Gibbs free-energy diagram for **c** OER of BHZ-48, **d** ORR of ZnHZ, CoHZ, and BHZ-48 with corresponding top views of the structures. Blue, gray, white, red, green, and orange balls represent the N, C, H, O, Co, and Zn atoms, respectively. **e** the calculated *d* band density of state for the transition metal atoms in ZnHZ, CoHZ, and BHZ-48. **f** Schematic illustration explaining change of metal-adsorbate interaction by altering the metal *d* band center (ε_*d*_). **g** Schematic representations of the electronic coupling between Co and Zn.

To decipher the underlying relevance of *V*_L_ on the material electrochemical behaviors, spectroscopic measurements and theoretical modeling of the two half reactions at air cathode are conducted. Three-coordinated BHZ-48 and four-coordinated BHZ-96 were processed to different potentials and investigated by EXAFS. As shown in Fig. 5, Co *K*-edges of BHZ-48 and BHZ-96 both shift to higher energy with increasing potential during OER, suggesting elevation in average oxidation associated with deprotonation of bridged OH* and OOH* on Co sites. The edge shift in BHZ-48 is larger than BHZ-96 within the same potential window, indicating better proton extraction from oxygen intermediates due to the presence of *V*_L_. When processed back to the open-circuit voltage, both energy and Co-N coordination condition show insignificant differences with pristine electrocatalysts, implying high reversibility of the deprotonation reaction in both BHZ-48 and BHZ-96. This can be explained by the self-healing feature of MOFs, which allows unrestricted DI ligands to substitute bridged OH^–^, followed by the recovery of Co coordination configurations of BHZ-48 and BHZ-96 to their pristine state^54^. Then, free-energy diagrams of the reaction mechanism are modeled based on density functional theory (DFT) to validate the correlation between the coordination structure of metal nodes and OER activity. As shown in Fig. 6a, the OER pathway involves four protons/electrons transferred steps and three intermediates of *OH, *O, and *OOH. In an ideal catalyst, the adsorption energy barrier is 2.46 eV for *OH to *OOH transformation (Δ*E*_*d*_ = ΔG_*OH_ – ΔG_*OOH_)^55^. As shown in Fig. 6b, it is clear that BHZ-48 is closer to this value compared to BHZ-96. The Gibbs free energy difference between BHZ-48 and BHZ-96 lead to different potential-determining step (PDS) of OER. The PDS for BHZ-96 is *OOH deprotonation into O_2_ molecule that requires a reaction free-energy barrier of 2.05 eV, whereas that for BHZ-48 is *OH deprotonation with a readily surmountable energy barrier of 1.75 eV (Fig. 6c and Supplementary Fig. 24). Consequently, the reduced PDS energy barrier accelerates the OER kinetics of BHZ-48, resulting in a 30 mV lower theoretical OER overpotential $(\eta _{OER}^t)$ than BHZ-96 (Fig. 6c). Furthermore, the electrochemical activation energies are extracted from OER Arrhenius plots and assessed to further corroborate that the *V*_L_ is a critical factor for intrinsic activity improvement (Supplementary Fig. 25). Comparatively, BHZ-48 exhibits a much smaller apparent barrier value of 12.2 kJ mol^−1^ than BHZ-96 (17.6 kJ mol^−1^), which aligns well with DFT simulations.

The influence of electronic state variations caused by Co-Zn alliance on ORR activity is also investigated by free-energy diagrams (Fig. 6d). In this case, the heterometallic BHZ-48 was compared with monometallic Zn ZIF and Co ZIF (denoted as ZnHZ, CoHZ, respectively). For ZnHZ, the initial oxygen activation step (transformation of O_2_ to *OOH) is the PDS owing to the weak interaction between active center and adsorbates. In comparison, CoHZ and BHZ-48 both encounter relatively low resistance during the oxygen activation step, and hence *OH desorption with large energy barriers is their PDS (Fig. 6d). Between the two, BHZ-48 exhibits lower OH* adsorption energy resulting in eased OH^−^ release (Supplementary Fig. 26). This trend follows the Sabatier principle, i.e., insufficient adsorption energy is adverse to progression of the subsequent reactions, while excessive adsorption would lead to difficulty in product desorption^56^. For an intuitive comparison, ORR overpotential ($\eta _{ORR}^t$) on each catalytic site was further calculated under the thermodynamic equilibrium potential of ORR at *U* = 1.23 V (Supplementary Fig. 27). The lowest $\eta _{ORR}^t$ is obtained in BHZ-48, which is due to its optimal adsorption/desorption behaviors established by fine-tuning of the electronic structure. Further insights are gained by examining the metal centers density of states (DOS), as the *d* band center (ε_*d*_) is highly correlated with the metal-adsorbate interaction. As shown in Fig. 6e and f, the upshift of ε_*d*_ is observed in BHZ-48 compared to ZnHZ, reflecting the positive influence of Co-Zn heterometallic alliance. The explanation for the coupling phenomenon is demonstrated using their electronic structure in Fig. 6g. Specifically, the valence electron configuration of Zn^II^ is 3*d*^10^ with fully occupied *d*-orbitals in the π-symmetry *t*_*2*_ (*d*_xy, xz, yz_) orbitals^57^. For this reason, electron repulsion is the dominating interaction between the bridging N and Zn. In contrast, the π-symmetry orbitals of Co^II^ in tetrahedral geometry with high-spin state are half-empty, which can interact with bridging N via π-donation. As a result, the cooperative effects within Co-Zn alliance enhance the π-donation interaction of Co-N by electron repulsion of Zn–N, promoting the partial charge transfer from Zn towards Co, and lowering the energy level of Co *d*-orbitals^37^.

## Discussion

At this point, the physicochemical and electrochemical properties of Co-Zn heterometallic ZIF has been characterized and analyzed. Their performance in ZABs is attributed to several critical factors that were endowed by its facile and versatile synthetic scenario. First, its hierarchical porosity introduced by the pyrolysis-avoided competing coordination strategy significantly relieves the electrolyte flooding issues that obstructs O_2_ diffusion in microporous ZIF, which has long been a major challenge associated with ZIF-based catalysts. The introduction of mesopores is caused by competing crystal growth between the original metal nodes with external cations in the substitution reaction. During which, although both Zn^II^ and Co^II^ can coordinate with DI ligands into isostructural crystalline framework, Zn^II^ with full *d*-orbital occupation has much weaker coordination capability than Co^II^, which gives rise to new Co-N coordination matrix and mesopores. The synthetic method can be extended to ZIFs containing other metal nodes, e.g., Fe^II^, Ni^II^, and Cu^II^, as indicated in Supplementary Fig. 28. The second efficacy of such strategy is the realization of controlled ligand editing and heteroatomic doping, which allows the generation of ligand vacancies and tuning of oxygen intermediates adsorption/desorption barriers to shift catalytic activity towards the vertex. These results establish an applicable direction to improve ZABs performance and other electrocatalysis systems by modulating ligand environment and heterometallic alliance. In addition, the self-healing feature of MOFs is conducive to repairing metal-ligand coordination environment that alleviates performance degradation from active metal sites dissociation^54^. It differs from the irreversible evolution of metal compounds upon electrocatalytic process^17^. This behavior originates from the relatively weaker strength of coordination bonds (<200 kJ mol^−1^) than ionic or metallic bonds (>700 kJ mol^−1^)^58,59^. Lastly, the heterocyclic aromatic ligand of BHZ-48 without unpaired electron can effectively suppress undesirable attack by free oxygen radicals, thereby promoting strong durability in battery cycling^27^.

In summary, a strategy to synchronously create hierarchical porosity and steer orbital state of metal nodes is designed for ZIFs. In the as-prepared BHZ-48, the orbital configuration of active sites is modulated by both the near-range interaction with ligand vacancies and the long-range interaction of Co-Zn alliance. As a result, the adsorption/desorption energy of reactive intermediates achieves an optimal state, where the energy barrier for potential-determining step is significantly lowered. When BHZ-48 is applied as air electrode in rechargeable ZABs, a charge–discharge voltage-gap of 0.8 V and a stable cyclability over 1250 h are achieved at 15 mA cm^−2^. Based on these evidences and fundamental understandings, this study showcases not only a strategy to design a conceptually unique electrocatalyst in the vast collections of pristine MOFs, but also a direction to steer oxygen electrochemistry for heterogeneous catalysis and other electrochemical energy storage systems.

## Methods

### Materials

dimethyl imidazole (99%, Analytical grade), zinc nitrite hexahydrate (99%, Analytical grade), zinc acetate tetrahydrate (99%, Analytical grade), cobalt nitrite hexahydrate (99%, Analytical grade), potassium hydroxide (85%, Analytical grade) and methanol (99.9%, anhydrous) were purchased from Sigma-Aldrich. Nafion^TM^ dispersion (5 wt.% in ethanol) was purchased from Ion Power. All chemicals were used without further purification. The deionized water (18 MΩ) was obtained from a Millipore System.

### Synthesis of ZnMZ electrode

In all, 0.40 mmol Zn(NO_3_)_2_·6H_2_O was first dissolved in 40 ml deionized water. The solution was then added to 40 ml deionized water containing 0.53 mmol dimethyl imidazole under ultrasonication. Subsequently, a piece of Ni foam (NF) was immersed into the mixed solution and transferred into a 100 ml Teflon vessel, which is then placed at 60 °C for 24 h under airtight conditions. After cooling to room temperature, the NF was obtained, washed by deionized water, and dried in ambient air.

### Synthesis of BHZ-12, BHZ-24, BHZ-48, BHZ-60, BHZ-72, BHZ-84, and BHZ-96 electrodes

The BHZ-12, BHZ-24, BHZ-48, BHZ-60, BHZ-72, BHZ-84, and BHZ-96 were all prepared using the same procedure, but with different cation-exchange reaction time of as-prepared ZnMZ to Co(NO_3_)_2_·6H_2_O from 12, 24, 48, 60, 72, 84, or 96 h. In a typical synthesis of BHZ-48, the as-prepared ZnMZ electrode was immersed into 25 ml methanol containing 1.00 mmol of Co(NO_3_)_2_·6H_2_O for 48 h at room temperature under airtight conditions. Finally, the NF was obtained, washed by deionized water, and dried in ambient air.

### Synthesis of FeHZ, NiHZ, and CuHZ

The FeHZ, NiHZ, and CuHZ samples were prepared using a similar procedure to that described above for BHZ-48, except that Co(NO_3_)_2_·6H_2_O were adjusted to Fe(NO_3_)_2_, Ni(NO_3_)_2_·6H_2_O, Cu(NO_3_)_2_·3H_2_O with same mole number.

### Synthesis of BMZ

In all, 0.12 mmol Zn(NO_3_)_2_·6H_2_O and 0.28 mmol Co(NO_3_)_2_·6H_2_O were first dissolved in 40 ml deionized water. The solution was then added to 40 ml deionized water containing 0.53 mmol dimethyl imidazole under ultrasonication. Subsequently, a piece of NF was immersed into the mixed solution and transferred into a 100 ml Teflon vessel, which is then reacted at 60 °C for 24 h under airtight conditions. After cooling to room temperature, the NF was obtained, washed by deionized water, and then dried in ambient air.

### Fabrication of Zn–air battery

Copper foil was used as current collector for the zinc anodes. The BHZ-48 was directly used as the air cathode. Commercial state-of-the-art 30 wt.% Pt/C and RuO_2_ catalysts with the same mass loading were measured and compared as the references. Homogeneous catalyst ink consisting of the nanocomposites, ionomer (Nafion solution, 5 wt.%) and ethanol was sprayed onto a nickel-based gas diffusion layer (Ion Power Inc., 35 BC) with a catalyst loading of 1 mg cm^–2^. The air cathode was then paired with a zinc plate anode and assembled in a battery prototype filled with solution of 6.0 M KOH and 0.20 M Zn(CH_3_COO)_2_. The flexible Zn–air battery was fabricated through layer-by-layer method, and assembled in the order of zinc foil, cellulose membrane, the BHZ-48 and the gas diffusion backing layer. The cellulose membrane was pre-wetted by 6 M KOH and 0.20 M Zn(CH_3_COO)_2_ solution before the battery assembly.

### Materials characterization

Transmission electron microscopy images were taken using a JEOL 2010F microscope operated at 120 kV. X-ray diffraction (XRD) patterns were obtained by a Bruker AXS D8 Advance powder X-ray diffractometer equipped with a Cu Kα radiation source (*λ* = 1.5406 Å) and a graphite monochromator. X-ray photoelectron spectroscopy (XPS) measurements were carried out by a Thermal Scientific K-Alpha XPS spectrometer. Wide-angle X-ray scattering (WAXS) images were acquired at Very Sensitive Elemental and Structural Probe Employing Radiation beamline of Canadian Light Source. The energy of X-ray beam used for WAXS is 8 keV. The X-ray Microdiffraction Analysis software was employed to integrate and obtain the synchrotron X-ray diffraction (SXRD) patterns. X-ray absorption spectroscopy (XAS) measurements on Co and Zn *K*-edge were performed in fluorescence mode at Canadian Light Source, Canada, using Soft X-ray Microcharacterization Beamline and Biological X-Ray Absorption Spectroscopy. Electrodes at different potential conditions were extracted from electrolyzer, sealed and measured immediately. All XAS data were processed using Athena and Artemis software packages. Thickness of the material was characterized by atomic force microscope (Vecco Dimension 3000). Electron-paramagnetic resonance was acquired at 77 K by a Bruker EMX spectrometer (2-mWmicrowave power, 9.4-GHz microwave frequency). Inductively coupled plasma optical emission spectrometer methodology was conducted on a Thermo Scientific iCAP 6500 duo optical emission spectrometer. Elemental analysis was performed on a Thermo Finnigan FLASH EA 1112 Series. The pore structure was investigated by N_2_ adsorption–desorption measurements on a Brunauer-Emmett-Teller surface area analyzer (Quantachrome Instruments QuadraSorb SI4) and a Barrett-Joyner-Halenda (BJH) model was used to obtain the pore-size distribution.

### Electrochemical measurement

The electrochemical measurements were performed in a three-electrode system with an electrochemical workstation (Biologic VMP-3) at ambient temperature and pressure. The three-electrodes system contains the as-fabricated electrodes, a graphite rod, and a reversible hydrogen electrode (RHE) as the working, counter and reference electrodes, respectively. To obtain the linear sweep voltammetry (LSV) curves at different rotating speeds, a rotating disc glassy carbon electrode (GC; 5 mm in diameter) was used as the working electrode. Acrylic tape (3 M) was used to secure the samples (such as BHZ-48 and Pt/C on nickel foam, 5 × 5 mm^2^) on the glassy carbon rotating disc electrode tip. Commercial Pt/C (30 wt.% Pt) and RuO_2_ catalysts were used as the reference materials. The catalyst ink was prepared by mixing 4 mg of commercial catalyst powder in 1 ml of ethanol containing 0.15 wt.% Nafion^TM^ dispersion, followed by ultrasonication for 1 h. Then 15 µl of the as-prepared ink was drop casted onto nickel foam to give a catalyst loading of 0.2 mg cm^–2^. All the measurements were carried out in 0.1 M KOH solution, where O_2_ or N_2_ gas was purged for 30 min before ORR or OER measurements, respectively. The LSV measurements were scanned from 1.0 to 0.1 V for ORR at different rotation speeds and from 1.0 to 1.8 V for OER at steady state with scan rate of 10 mV s^–1^. ORR and OER polarization curves were corrected by IR-compensation in 0.1 M KOH solution, where the resistance is determined by the high-frequency intercept of the Nyquist plot acquired from electrochemical impedance spectroscopy (EIS). The EIS was performed with a frequency ranging from 100 kHz to 0.1 Hz with potential amplitude of 50 mV. Capacitive background currents were subtracted for ORR and OER polarization curves during cyclic voltammetry (CV) measurements in N_2_-saturated KOH solution. The stability studies on the half-cell reactions (ORR and OER separately) was primarily assessed by chronoamperometric and chronopotentiometric measurements at a given constant potential or a constant current for 300 h. Galvanostatic discharge and charge cycling of the Zn–air batteries was performed through a recurrent galvanic pulse method at current densities of 15 mA cm^–2^ with 10 h per cycle. These galvanostatic charge/discharge curves were recorded using a LAND battery testing station (CT2001A) at room temperature. Polarization data was collected using the galvanodynamic method at a scan rate of 1.0 mA s^–1^ with cut-off voltages of 0.5 V for the discharge curves and 2.5 V for the charge curves.

### Computational details

All the spin-polarized computations were performed by using Vienna ab initio simulation package (VASP). The ion-electron interactions were described by the projector augmented wave method and the general gradient approximation in the Perdew–Burke–Ernzerhof (PBE) form was used. During the structure relaxation, the convergence criterion was set to 0.05 eV Å^−1^ and 10^−5^ eV for the residual force and energy, respectively. The models we used to simulate the BHZ-96, BHZ-48, ZnHZ and CoHZ. For BHZ-48, ZnHZ and CoHZ, the metal sites are terminated by OH group, owing to the high surface free energy of the metal atoms. To avoid the interaction between two periodic units, a vacuum space of 20 Å was employed.

The OER may proceed through the following elementary steps (Eqs. (1–4)), which are usually employed to investigate the electrocatalysis of the OER on various materials:

1 $${\mathrm{OH}}^-\left( {{\mathrm{aq}}} \right) + \ast \to \ast {\mathrm{OH}} + {\mathrm{e}}^-$$

2 $${\mathrm{OH}}^-\left( {{\mathrm{aq}}} \right) + \ast {\mathrm{OH}} \to \ast {\mathrm{O}} + {\mathrm{H}}_2{\mathrm{O}} + {\mathrm{e}}^-$$

3 $${\mathrm{OH}}^-\left( {{\mathrm{aq}}} \right) + \ast {\mathrm{O}} \to \ast {\mathrm{OOH}} + {\mathrm{e}}^-$$

4 $${\mathrm{OH}}^-\left( {{\mathrm{aq}}} \right) + \ast {\mathrm{OOH}} \to {\mathrm{O}}_2 + {\mathrm{H}}_2{\mathrm{O}} + {\mathrm{e}}^- + \ast$$

The ORR occurring through elementary steps takes the reverse direction of OER (Eqs. (5–8)):

5 $${\mathrm{O}}_2 + {\mathrm{H}}_2{\mathrm{O}} + {\mathrm{e}}^- + \ast \to \ast {\mathrm{OOH}} + {\mathrm{OH}}^-\left( {{\mathrm{aq}}} \right)$$

6 $$\ast {\mathrm{OOH}} + {\mathrm{e}}^- \to {\mathrm{OH}}^-\left( {{\mathrm{aq}}} \right) + \ast {\mathrm{O}}$$

7 $$\ast {\mathrm{O}} + {\mathrm{H}}_2{\mathrm{O}} + {\mathrm{e}}^- \to \ast {\mathrm{OH}} + {\mathrm{OH}}^-\left( {{\mathrm{aq}}} \right)$$

8 $$\ast {\mathrm{OH}} + {\mathrm{e}}^- \to {\mathrm{OH}}^-\left( {{\mathrm{aq}}} \right) + \ast$$

The free-energy change (Δ*G*) of each elementary reaction was calculated as

9 $${\Delta}G = {\Delta}E + {\Delta}E_{{\mathrm{ZPE}}} - T{\Delta}S$$

In Eq. (9), Δ*E*, *E*_ZPE_, *T*, and *S* is the reaction energy difference, zero-point energies, temperature and entropy, respectively.

## Supplementary information

Supplementary Information

## Data Availability

The data that support the findings of this study are available from the corresponding author upon reasonable request.
