# Supplementary material for: d-Orbital steered active sites through ligand editing on heterometal imidazole frameworks for rechargeable zinc-air battery
Source: Nat Commun. 2020 Nov 17;11:5858. doi: 10.1038/s41467-020-19709-6 (PMC7673988; doi:10.1038/s41467-020-19709-6)
Supplement: Supplementary file 1 — Supplementary Information [file 41467_2020_19709_MOESM1_ESM.pdf]

Supplementary Information

***d*-Orbital steered active sites through ligand editing on heterometal imidazole frameworks for rechargeable zinc-air battery**

Jiang et al.

Supplementary Figures

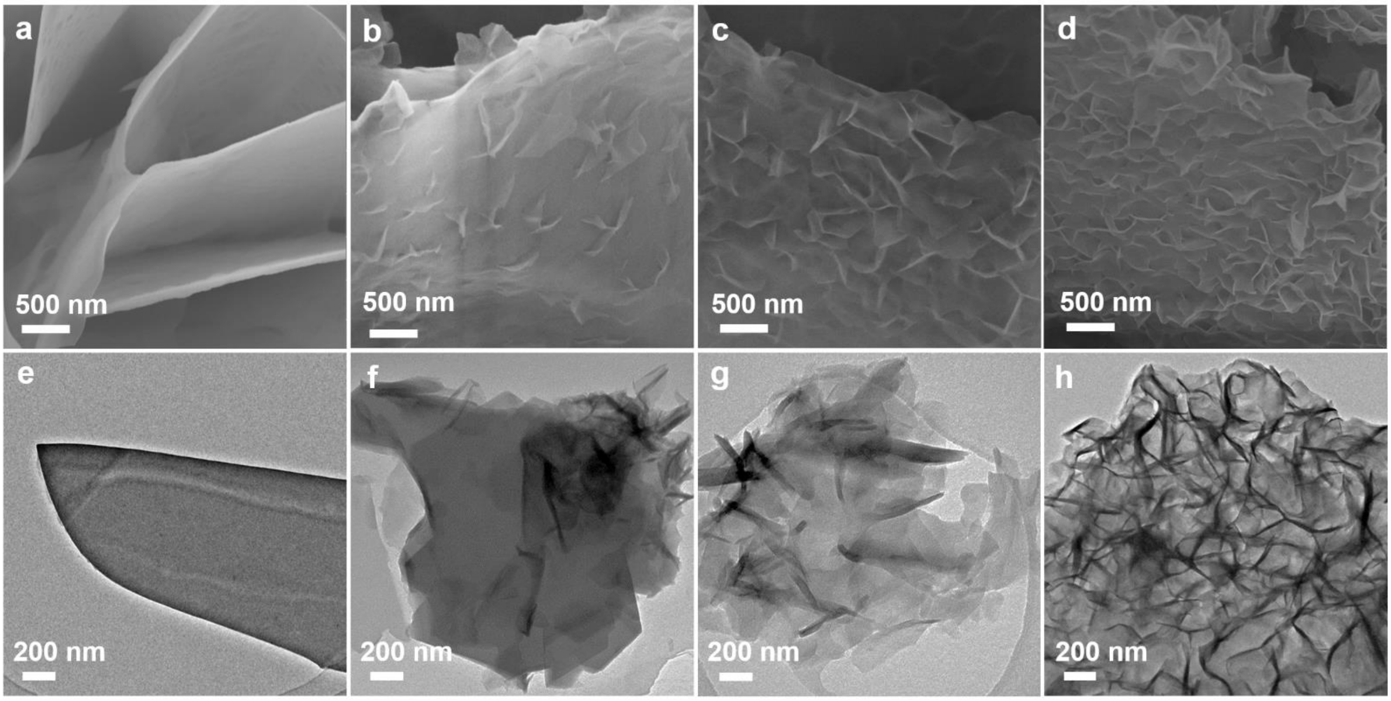

Supplementary Figure 1. Morphology of BHZ series. a-d) SEM, and e-h) TEM images of the (a, e) ZnMZ, (b, f) BHZ-12, (c, g) BHZ-24, and (d, h) BHZ-96.

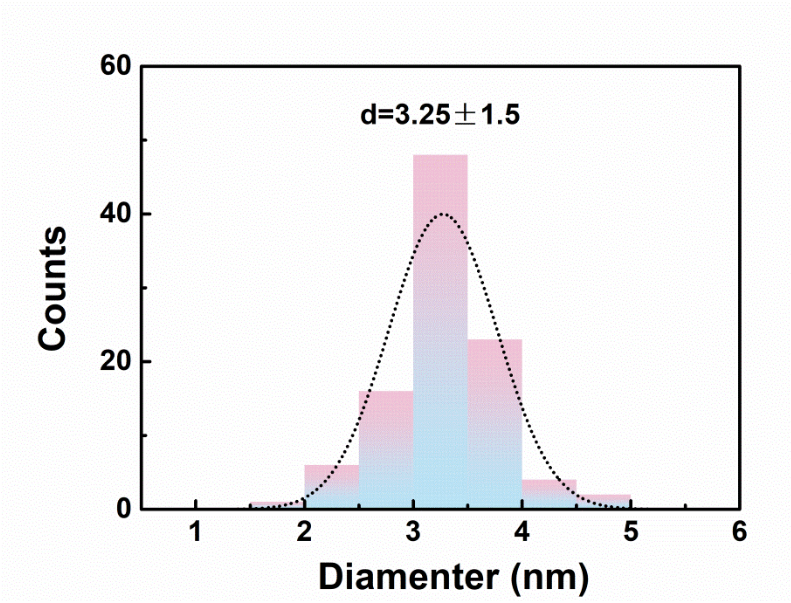

Supplementary Figure 2. Lateral length distribution of single sheet for ZnMZ measured from SEM image.

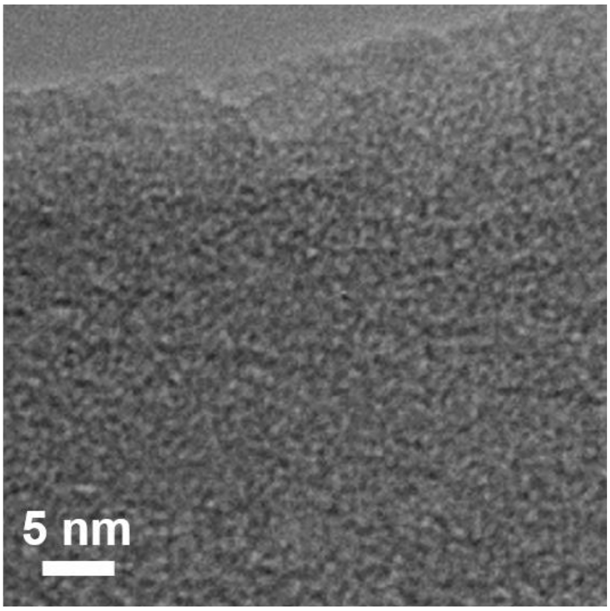

Supplementary Figure 3. High-resolution TEM image of ZnMZ.

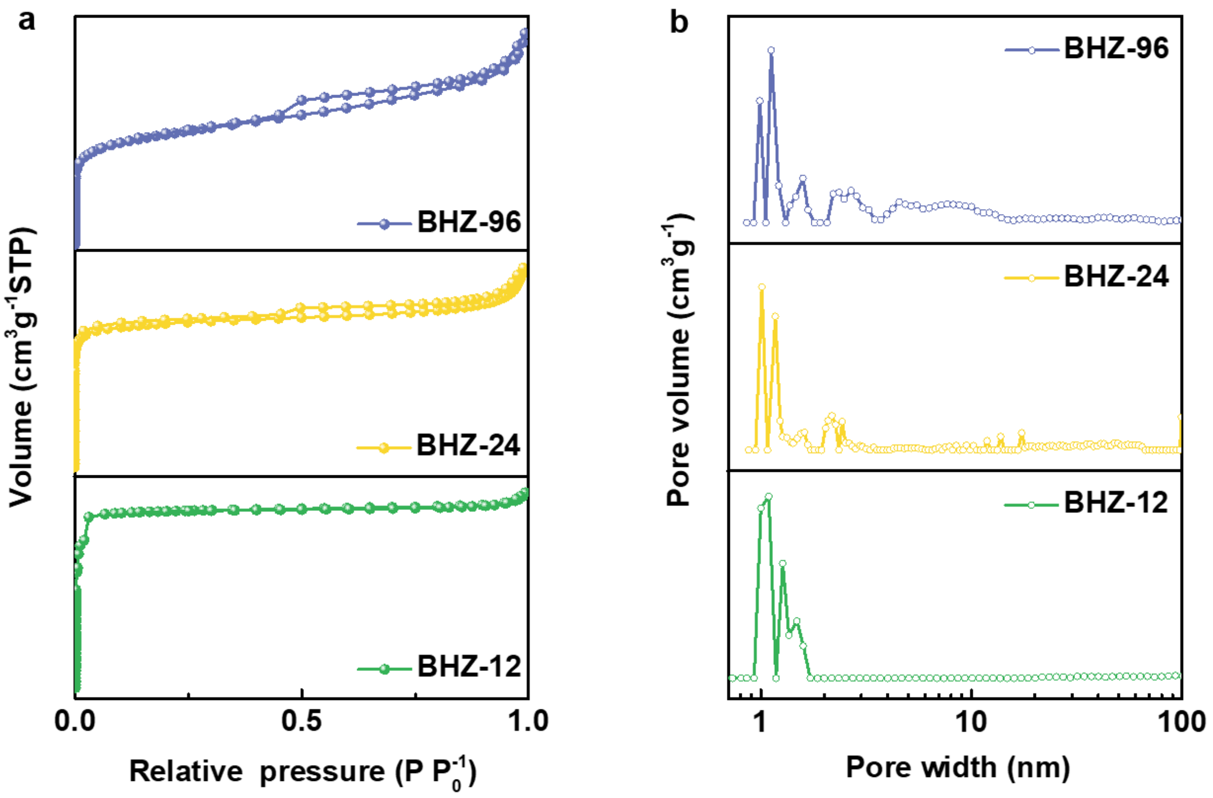

Supplementary Figure 4. Brunauer–Emmett–Teller (BET) surface area analysis. a) N_2 adsorption-desorption isotherms, b) pore-size distribution of BHZ-12, BHZ-24, and BHZ-96.

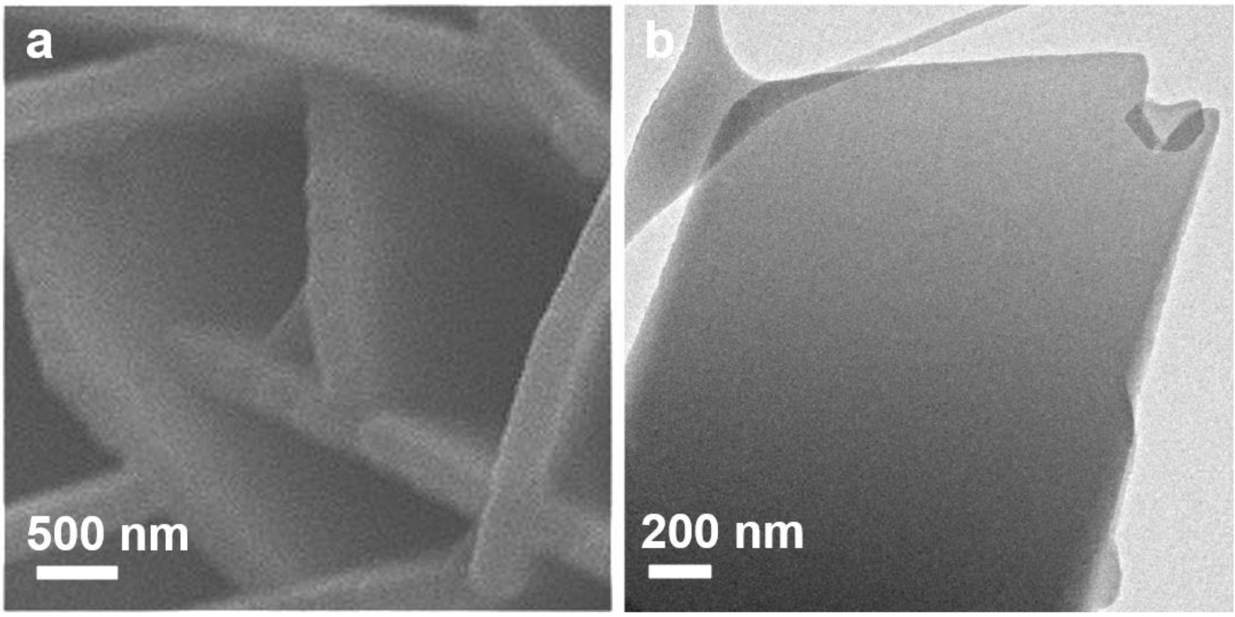

Supplementary Figure 5. Morphology of BMZ. a) SEM and, b) TEM images of BMZ.

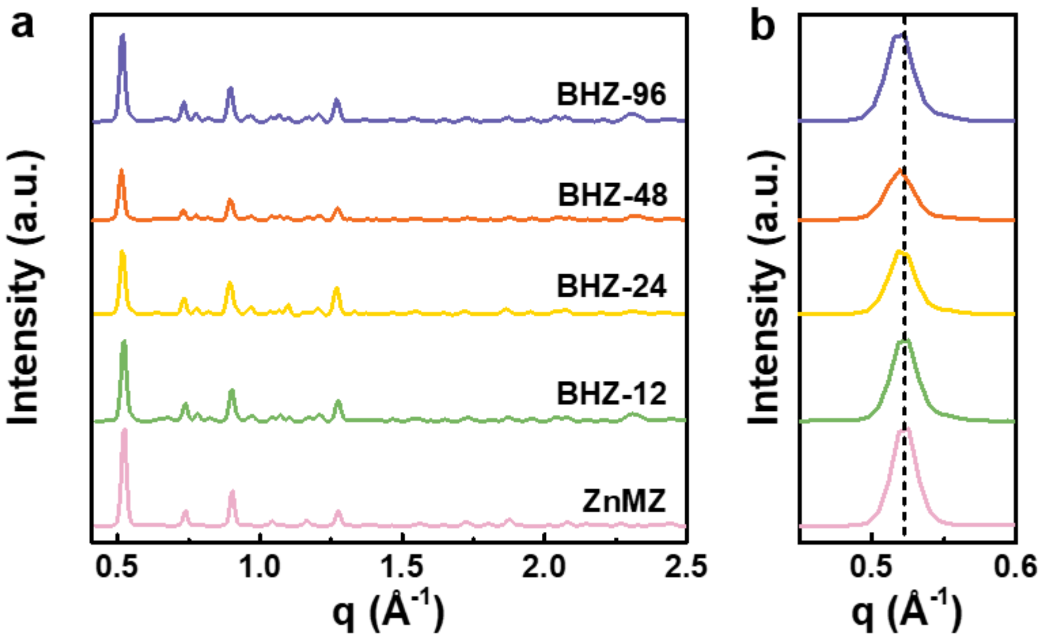

Supplementary Figure 6. Structural characterization. a) The integrated WAXS data from Figure 1j, and b) amplified peaks of BHZ-48 and comparison samples.

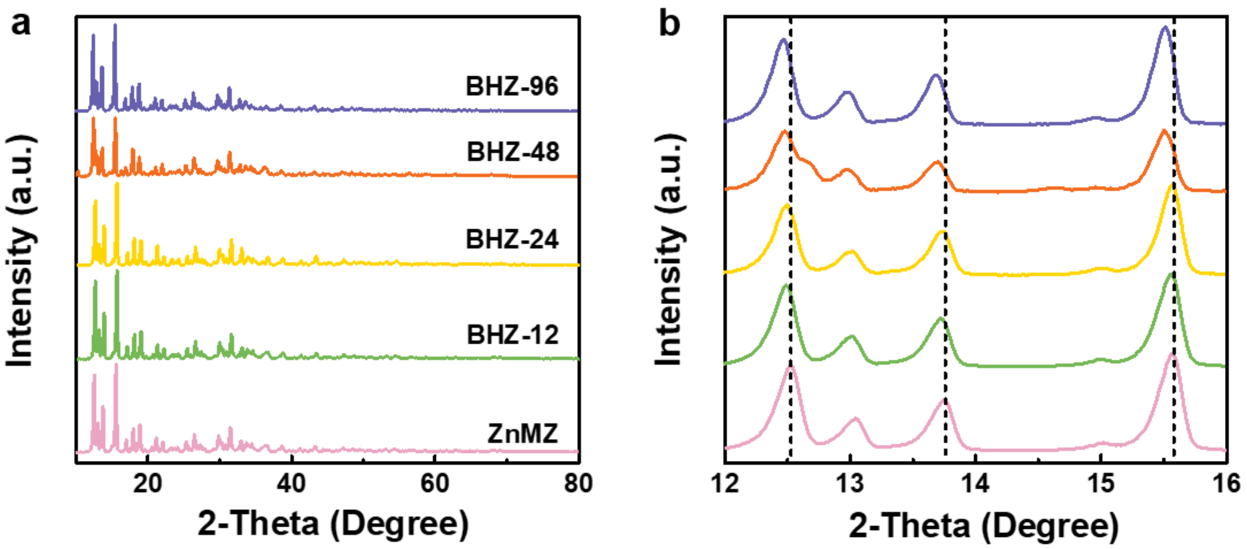

Supplementary Figure 7. XRD characterization. a) XRD patterns and b) amplified XRD peaks of BHZ-48 and comparison samples.

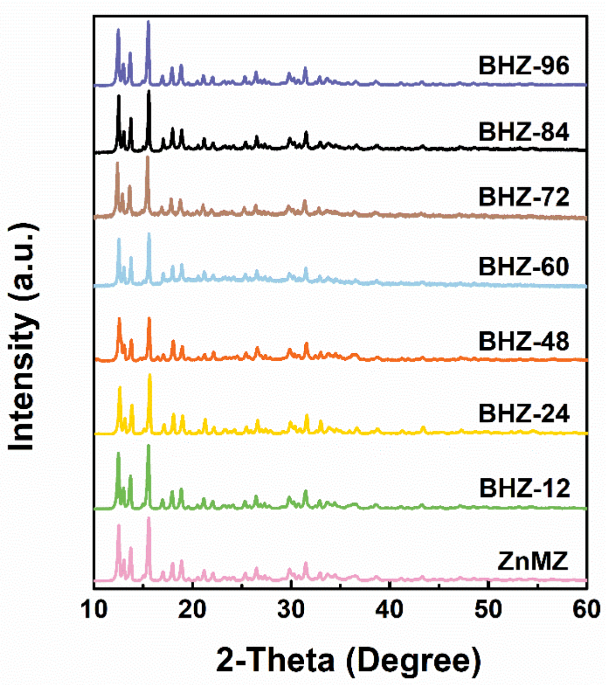

Supplementary Figure 8. Amplified XRD patterns of ZnMZ and BHZ treated by cation-substituion for differnet length of time. As shown, BHZ-(60, 72, 84) are more crystalline than BHZ-48, suggesting that the latter have substantial amount of crystal defects, leading to reduction in long-range order of the solid-state lattice.

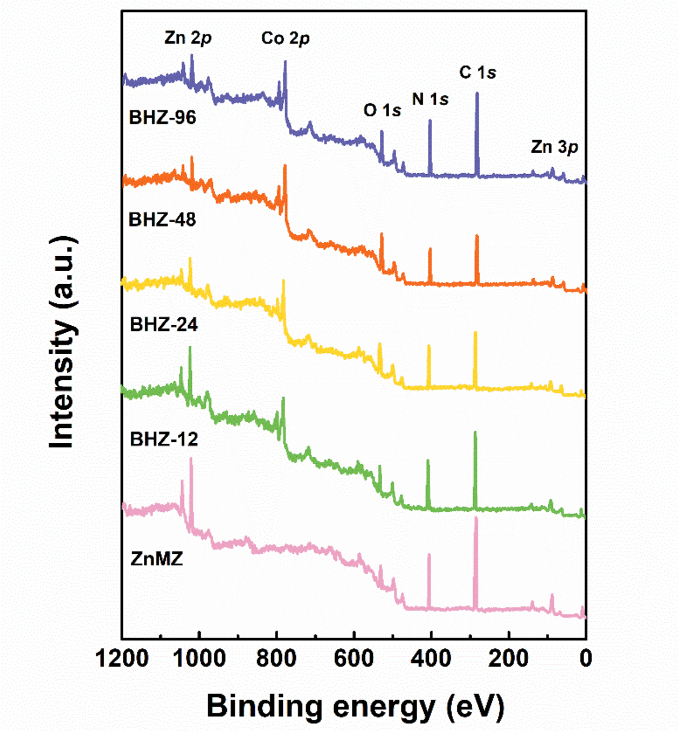

Supplementary Figure 9. XPS survery spectra of BHZ-48 and comparison samples.

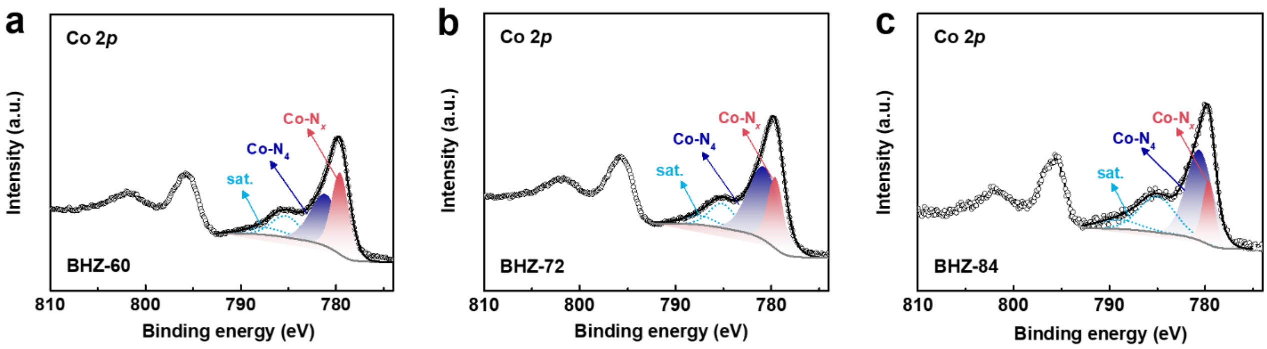

Supplementary Figure 10. XPS characterization. High-resolution Co *2p* XPS spectra of BHZ-60, BHZ-72 and BHZ-84.

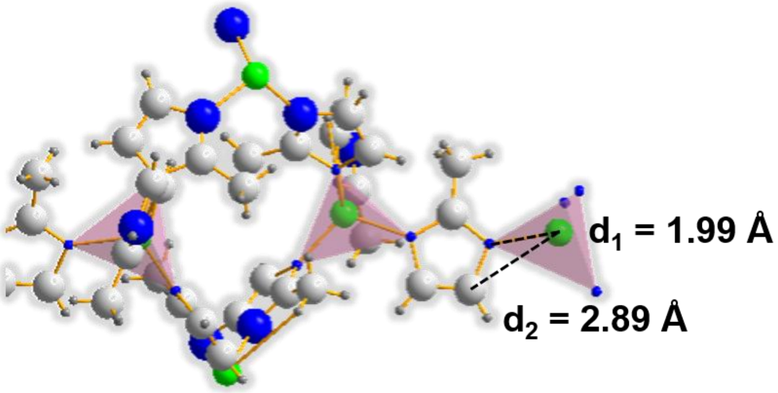

Supplementary Figure 11. Schematic illustration of the Co coordination environment, with their bond length determined by Diamond calculations. The coordination shells of Co-N and Co-C are marked with 1 and 2, respectively. Blue, light grey, dark grey, and green balls represent the N, C, H, and Co atoms, respectively.

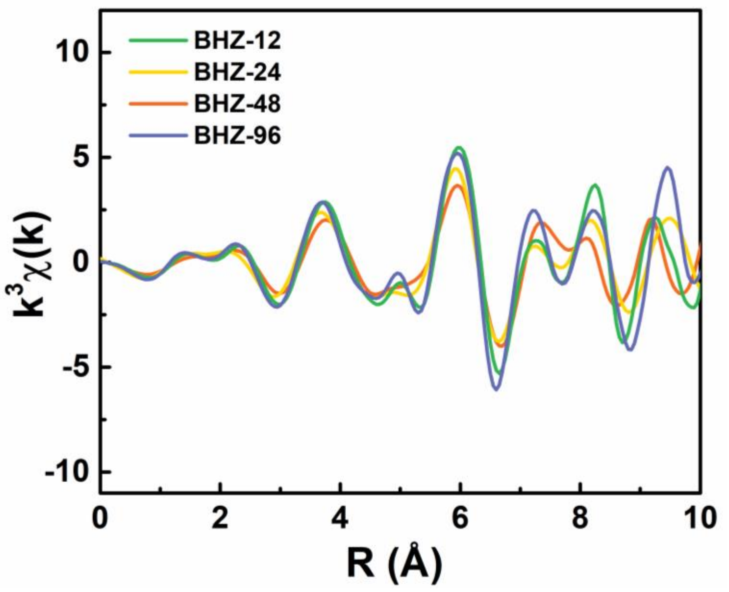

Supplementary Figure 12. Co *K*-edge extended XANES oscillation functions $k^3\chi(k)$.

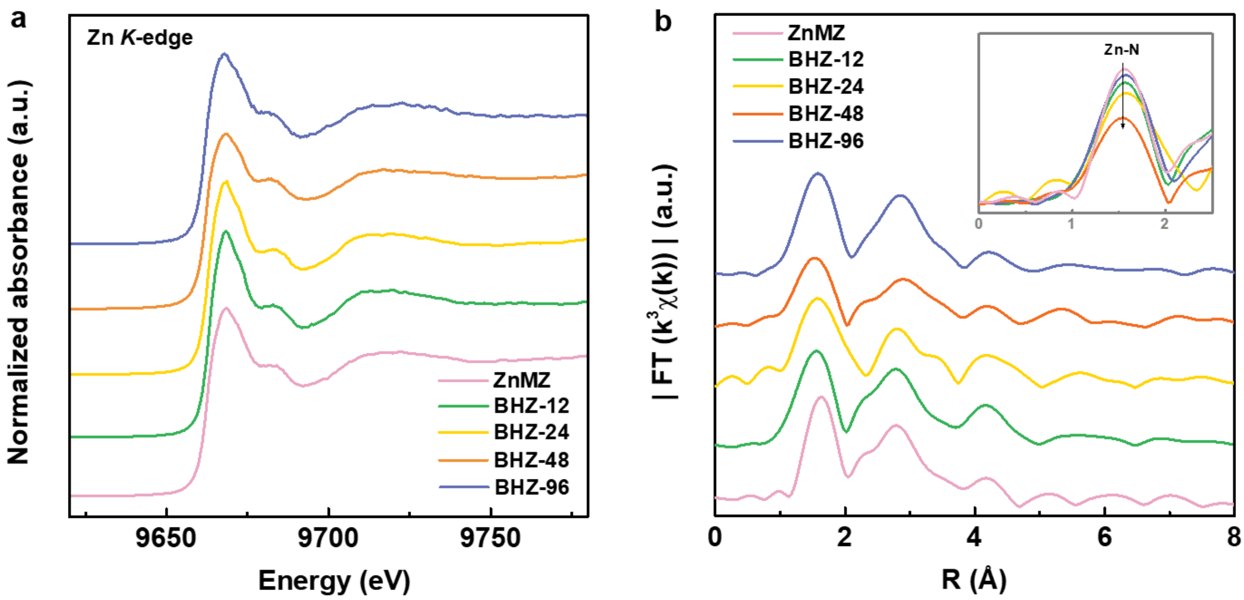

Supplementary Figure 13. XAS characterization. a) Zn *K*-edge XANES spectra, b) R-space Zn *K*-edge EXAFS spectra of the different samples.

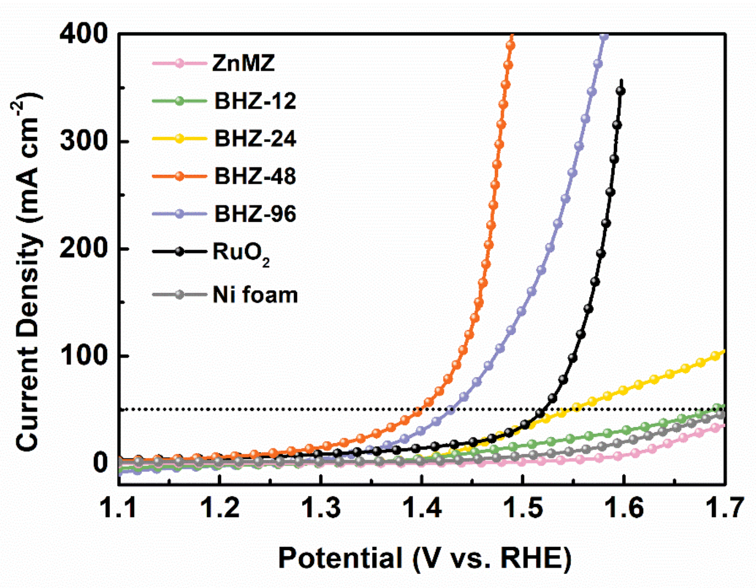

Supplementary Figure 14. OER LSV curves of bare Ni foam compared to catalysts grown on Ni foam electrodes. The bare Ni foam shows much higher OER overpotential than the catalyst-loaded counterparts, except for ZnMZ, which is due to almost complete coverage of the OER-inert Zn-based ZIF layer over the Ni foam.[1, 2]

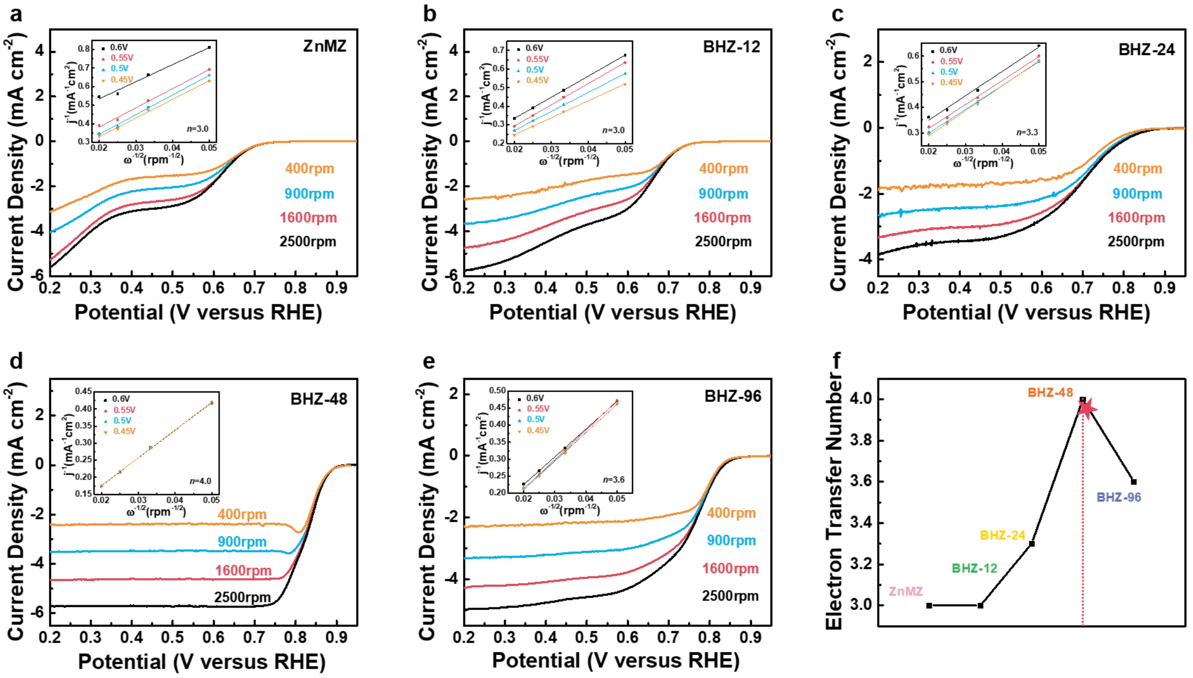

Supplementary Figure 15. Comparison of ORR activity for BHZ series. a-e) ORR polarization curves for BHZ-48 and comparison samples at various rotation speed (inset: Koutecky-Levich plot obtained at different potentials). f) Electron transfer number comparison of BHZ-48 and comparison samples.

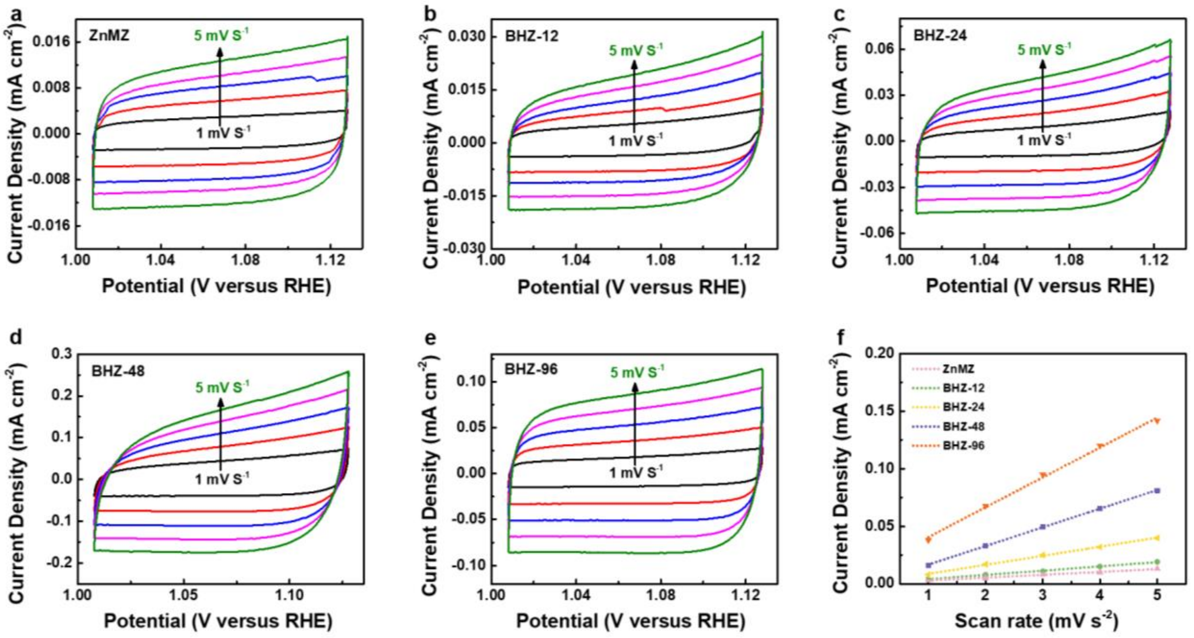

Supplementary Figure 16. Cyclic voltammetry (CV) curves and double-layer capacitance (C_{dl}). a-e) CV curves of BHZ-48 and comparison samples. All the CV curves are obtained in nitrogen-saturated 0.1 M KOH solution. f) Plots of current densities as a function of scan rates. The linear fitting slopes are used to determine their respective C_{dl} that corresponds with the electrochemical active surface area.

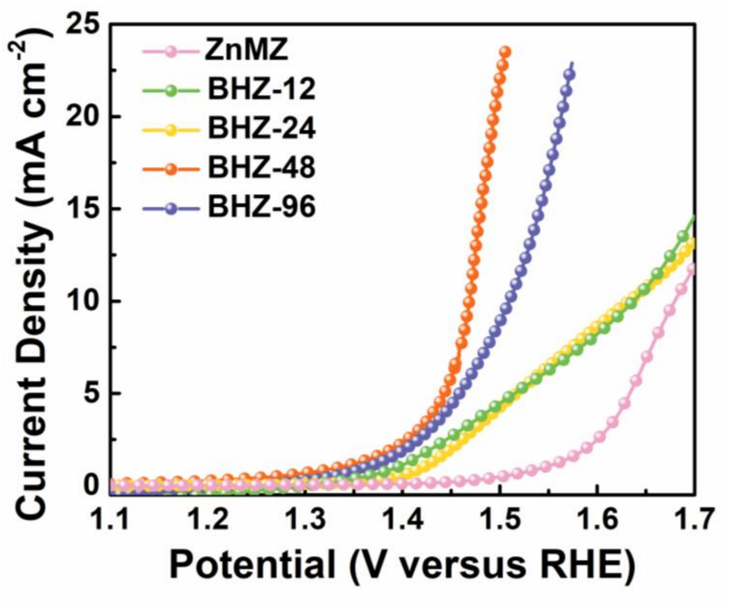

Supplementary Figure 17. LSV curves of the electrodes normalized by electrochemical active surface area.

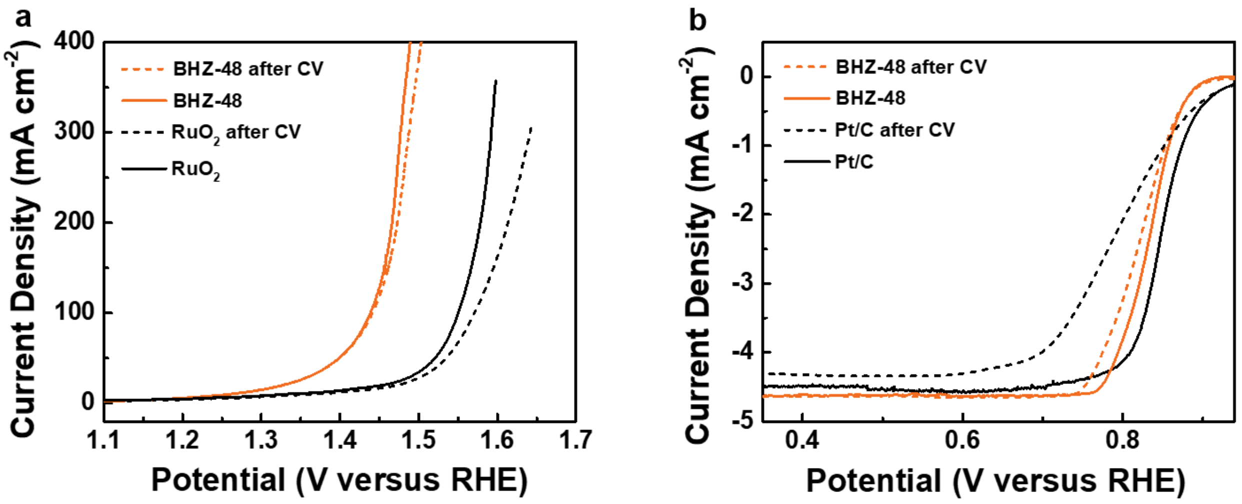

Supplementary Figure 18. OER and ORR stability of catalysts. a) OER activities of BHZ-48 and RuO_2 before and after 3000 cycles of CV scans between the voltage of 0.6 and 1.7 V in O_2-saturated 0.1 M KOH solution at 50 mV s^{-1}. b) ORR activities of BHZ-48 and Pt/C before and after 3000 cycles of CV between 0.6 and 1.7 V in O_2-saturated 0.1 M KOH solution at 50 mV s^{-1}.

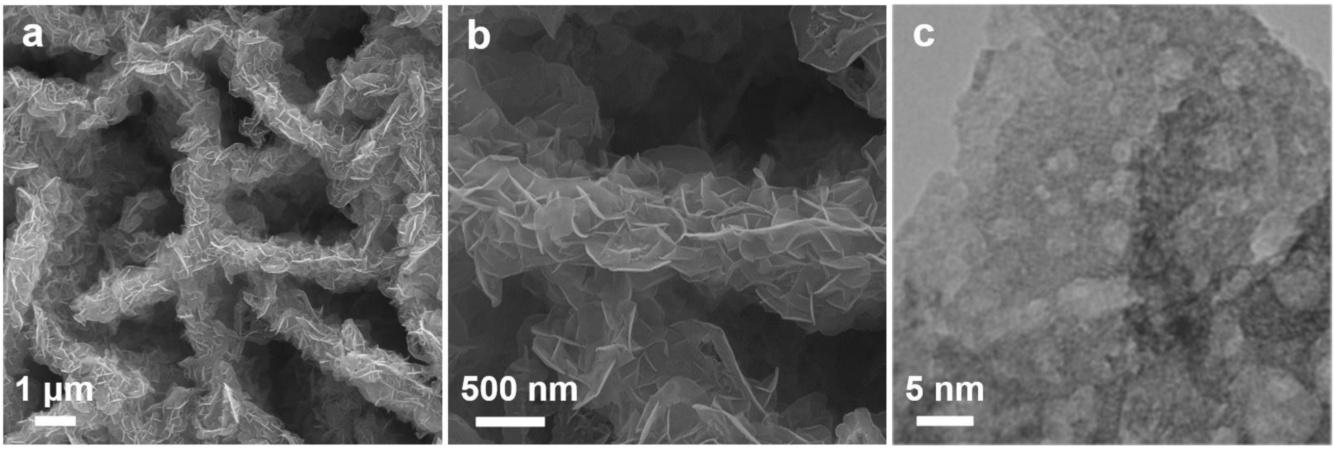

Supplementary Figure 19. Morphology of BHZ-48 after stability test. a, b) SEM and c) TEM images.

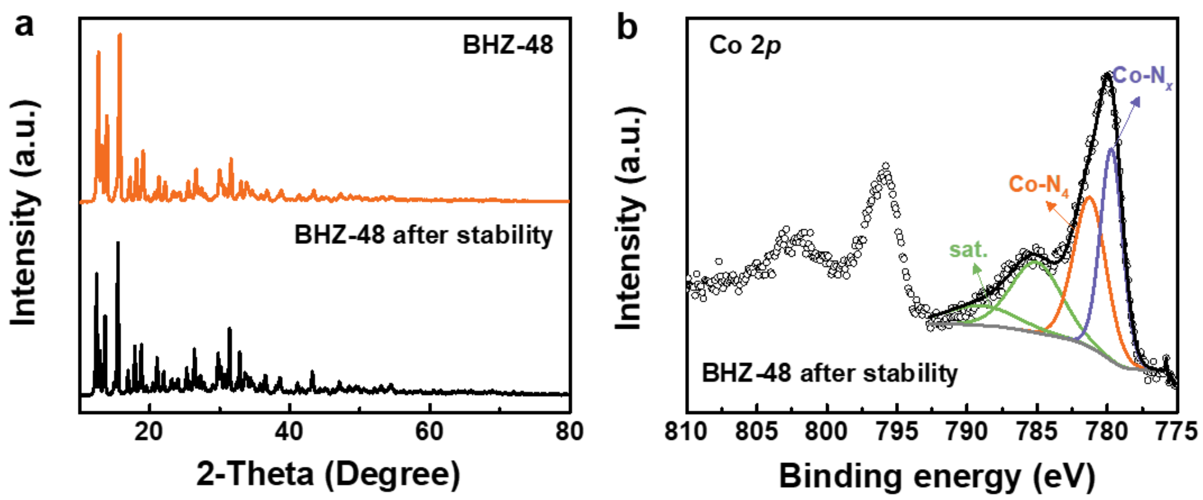

Supplementary Figure 20. Physical characterization of BHZ-48 after stability test. a) XRD patterns and b) high-resolution Co 2*p* XPS spectrum.

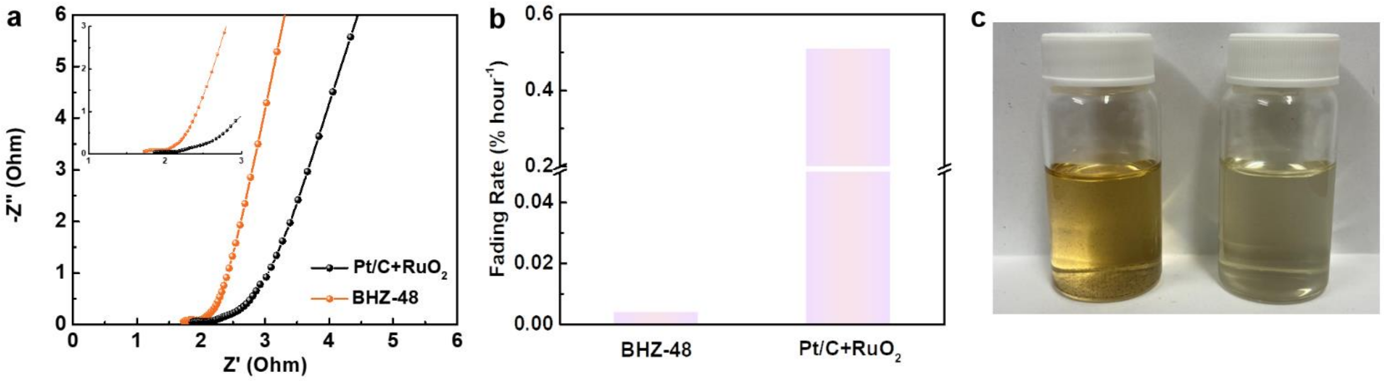

Supplementary Figure 21. Electrochemical and physical characterization. a) Nyquist plots of BHZ-48 and Pt/C+RuO_2 electrodes. b) Comparison of battery fading rate of BHZ-48 and Pt/C+RuO_2 electrodes. The fading rate is calculated by dividing the change in charge/discharge voltage with battery cycling time. c)The color changes of electrolyte after galvanostatic cycling stability with Pt/C+RuO_2 (left) and BHZ-48 (right).

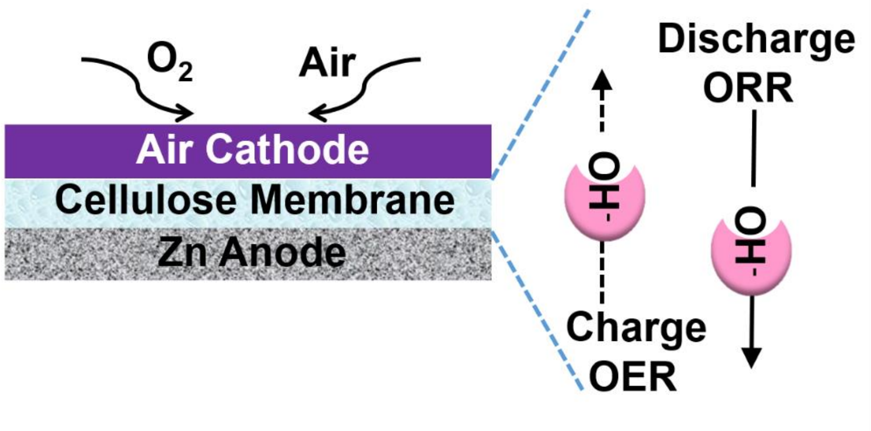

Supplementary Figure 22. Schematic illustration of the flexible Zn-air battery configuration.

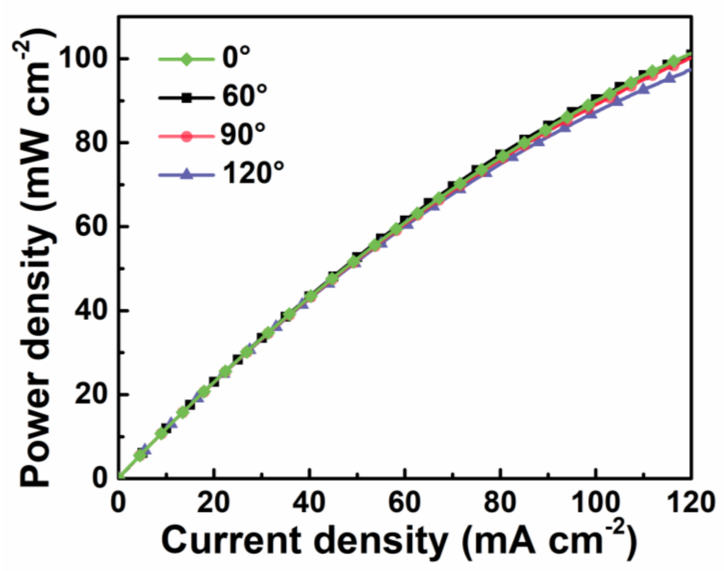

Supplementary Figure 23. The power density plots of the flexible Zn–air battery prepared using the BHZ-48 electrode under different bending angles.

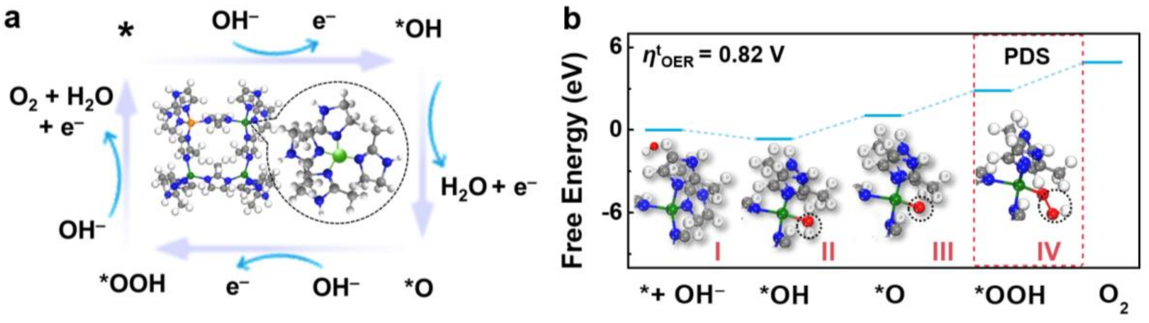

Supplementary Figure 24. DFT simulations and proposed mechanism illustration. a) Proposed OER catalytic mechanisms for BHZ-96. b) Gibbs free-energy diagram for OER of BHZ-96.

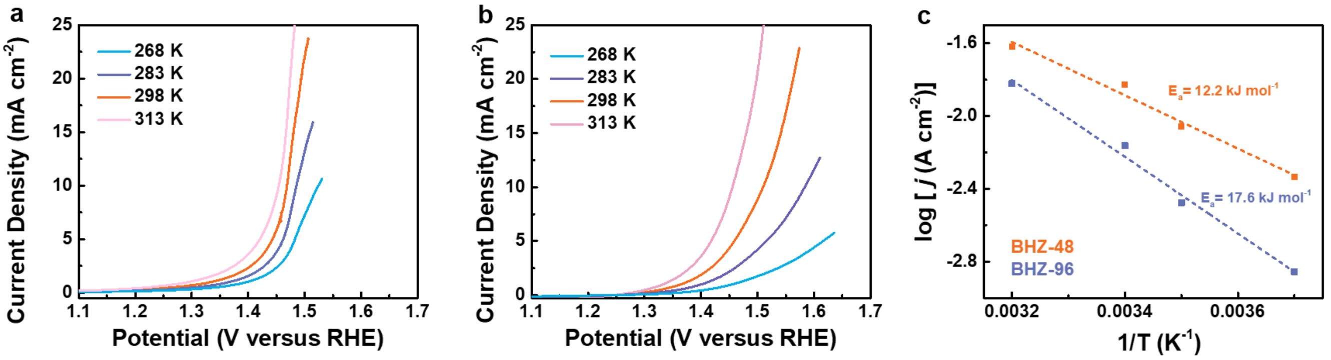

Supplementary Figure 25. Performance of BHZ-48 and BHZ-96 in three-electrode configuration. OER polarization curves normalized by electrochemical active surface area of a) BHZ-48 and b) BHZ-96 electrode tested at various temperatures. d) Log j versus $1/T$ and the fitted plot. The electrochemical activation energy (E_a) for OER can be estimated by the Arrhenius relationship:

$$\log j = \frac{-E_a}{ln\,10*R*T} + const \quad \text{(Supplementary Equation 1)}$$

where j is the current density at η = 300 mV, R is the universal gas constant (8.314 J K^{-1} mol^{-1}), T is the temperature. The E_a can be extracted from the slope of Arrhenius plot.[3]

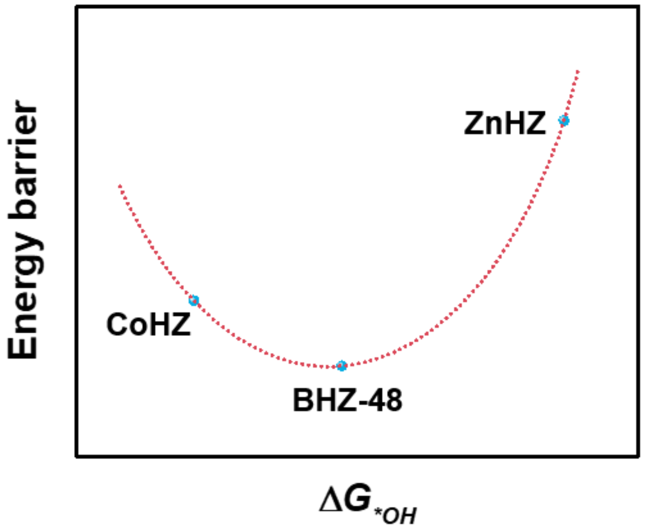

Supplementary Figure 26. The comparison of energy barrier of PDS and ΔG_{*OH} for ZnHZ, CoHZ, BHZ-48.

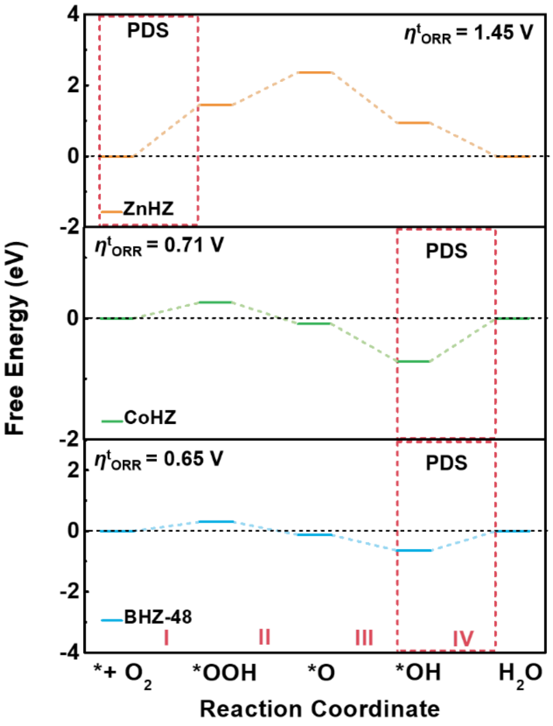

Supplementary Figure 27. Free energy diagram of ORR at U = 1.23 V on ZnHZ, CoHZ, and BHZ-48.

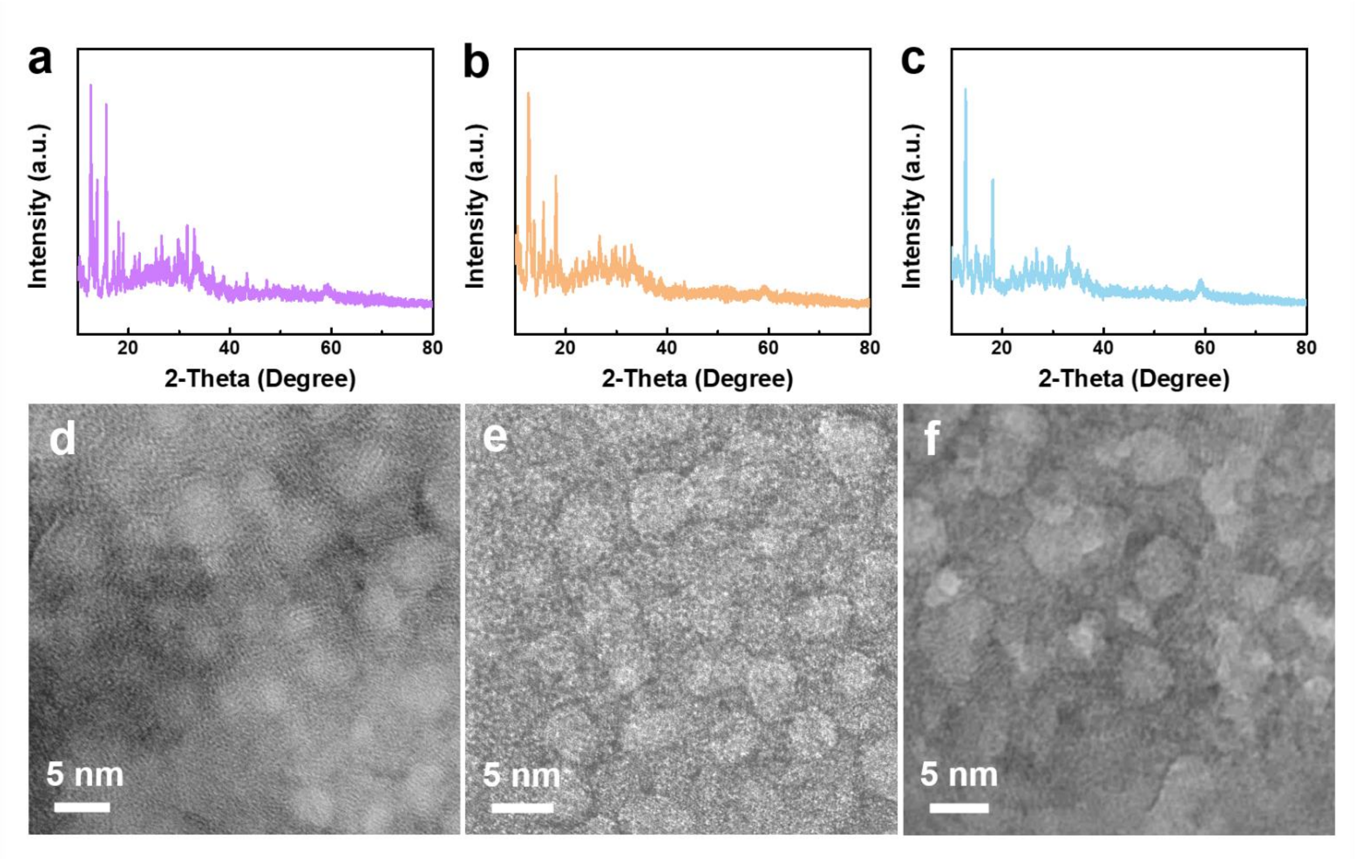

Supplementary Figure 28. Structural and morphological characterization. a-c) XRD patterns and d-f) TEM images of (a, d) FeHZ, (b, e) NiHZ, (c, f) CuHZ.

Supplementary Tables

Supplementary Table 1. Brunauer-Emmett-Teller (BET) specific surface area (S_{BET}) of as-prepared ZIFs.

	ZnMZ	BHZ-12	BHZ-24	BHZ-48	BHZ-96
S_{BET} (m^2g^{-1})	503	491	451	429	434

The BHZ-24, BHZ-48, and BHZ-96 possessed similar S_{BET}, which are relatively lower than those of the ZnMZ and BHZ-12, due to generation of additional mesopores.[4, 5]

Supplementary Table 2. Elemental analysis (EA) and inductively coupled plasma atomic emission spectroscopy (ICP-AES) results of as-prepared ZIFs.

	C	N	O	Zn	Co
ZnMZ	62.2	24.1	4.7	8.3	0
BHZ-12	61.5	23.7	5.7	4.1	4.2
BHZ-24	59.5	22.5	9.0	3.2	5.1
BHZ-48	57.4	19.2	14.5	2.5	5.7
BHZ-96	62.1	24.3	4.6	2.4	5.7

The ICP-AES is used for determining the percentage of metal in the ZIFs samples. Besides, the C, N and O content of ZIFs are determined by EA, which is closed to that of the XPS survey spectra.

Supplementary Table 3. Co *K*-edge EXAFS data fitting results of as-prepared ZIFs.

	Shell	CN	R (Å)	σ^2 (10^{-3} Å^2)	R-factor
BHZ-12	Co-N	4.0	2.11	4.2	0.0015
BHZ-24	Co-N	3.6	2.10	5.6	0.0018
BHZ-48	Co-N	2.9	2.10	7.0	0.0014
BHZ-96	Co-N	4.3	2.09	4.0	0.0021

CN is the coordination number; R is interatomic distance (the bond length between Co central atoms and surrounding coordination atoms); σ^2 is Debye-Waller factor (a measure of thermal and static disorder in absorber-scattered distances).

Supplementary Table 4. Oxygen electrode activities of as-prepared catalysts for ORR.

		ZnMZ	BHZ-12	BHZ-24	BHZ-48	BHZ-96
ORR	$E_{on\text{-}set}$ [a] (V)	0.75	0.77	0.86	0.90	0.87
	$E_{half\text{-}wave}$ (V)	0.63	0.67	0.73	0.84	0.78
	J_L [b] (mA cm^{-2})	NA	NA	-3.0	-4.6	-4.0

a) Onset potential was measured at -0.01 mA cm^{-2}.

b) Due to the testing manners, the limiting current density in this article is slightly less than common values reported in literature.

Supplementary Table 5. Tafel slope of as-prepared catalysts for OER and ORR.

Tafel slope ($mV\ dec^{-1}$)	ZnMZ	BHZ-12	BHZ-24	BHZ-48	BHZ-96
**OER**	374	355	349	80	177
**ORR**	93	89	122	45	76

Supplementary Table 6. Comparison of reported rechargeable zinc-air batteries.

Catalyst	Discharge current density (mA cm^{-2})	Average discharge working potential (V)	Total discharge time (h)	Cumulative energy density (mWh cm^{-2})	Reference
NPMC-1000	2	1.30	50	130	6
c-$CoMn_2$/C	10	1.25	8.5	106.25	7
CCO@C	2	1.30	40	104	8
$Ni_{6/7}Fe_{1/7}$-OH-6/CNT	15	1.20	12.5	225	9
NCNT/CoO-NiO-NiCo	20	1.15	8.5	195.5	10
Co_4N/CNW/CC	10	1.16	68	788.8	11
Co_3O_4/NPGC	5	1.15	41.5	238.63	12
CoO/N-CNT	20	1.25	100	2500	13
NGM-Co	2	1.00	36	72	14
La_2O_3/Co_3O_4/MnO_2-CNT	50	1.10	10.5	2300	15
Ni_3FeN/NRGO	10	1.17	15	175.5	16
3DOM-Co@TiO_xN_y	20	1.18	150	3540	17
Hybrid nanosheets	6	1.20	12.5	90	18
NCNF-1000	10	1.10	41.5	456	19
BHZ-48	15	1.18	1250	22125	This work

All average discharge working potential (V) is obtained by the reported rechargeable zinc-air profiles. The used energy density (E_{used}) calculated by the multiply of discharge current density (I_{dis}), average discharge working potential (V_{avg}) and total discharged time (T_{dis}).

$E_{used} = I_{dis} \times V_{avg} \times T_{dis}$ (Supplementary Equation 2)

Supplementary References

1. Sun, S., et al. Switch of the rate-determining step of water oxidation by spin-selected electron transfer in spinel oxides. *Chem. Mater.* **31**, 8106-8111 (2019).
2. Li, H., et al. Metal–oxygen hybridization determined activity in spinel-based oxygen evolution catalysts: A case study of $ZnFe_{2-x}Cr_xO4$. *Chem. Mater.* **30**, 6839-6848 (2018).
3. Zhang, J., *et al.* A highly sensitive breathable fuel cell gas sensor with nanocomposite solid electrolyte. *InfoMat* **1**, 234-241 (2019).
4. Asadullah, M., Rahman, M. A., Motin, M. A., Sultan, M. B. Preparation and adsorption studies of high specific surface area activated carbons obtained from the chemical activation of jute stick. *Adsorption Science & Technology* **24**, 761-770 (2006).
5. Dai, F., et al. Bottom-up synthesis of high surface area mesoporous crystalline silicon and evaluation of its hydrogen evolution performance. *Nat. Commun.* **5**, 3605 (2014).
6. Zhang, J., Zhao, Z., Xia, Z., Dai, L. A metal-free bifunctional electrocatalyst for oxygen reduction and oxygen evolution reactions. *Nat. Nanotech.* **10**, 444-452 (2015).
7. Li, C., Han, X., Cheng, F., Hu, Y., Chen, C., Chen, J. Phase and composition controllable synthesis of cobalt manganese spinel nanoparticles towards efficient oxygen electrocatalysis. *Nat. Commun.* **6**, 7345 (2015).
8. Wang, X., *et al.* Electrospun thin-walled $CuCo_2O_4$@C nanotubes as bifunctional oxygen electrocatalysts for rechargeable Zn–air batteries. *Nano Lett.* **17**, 7989-7994 (2017).
9. Wang, T., *et al.* NiFe (O_{xy}) hydroxides derived from NiFe disulfides as an efficient oxygen evolution catalyst for rechargeable Zn-air batteries: the effect of surface S residues. *Adv. Mater.* **30**, 1800757 (2018).
10. Liu, X., Park, M., Kim, M. G., Gupta, S., Wu, G., Cho, J. Integrating NiCo alloys with their oxides as efficient bifunctional cathode catalysts for rechargeable zinc-air batteries. *Angew. Chem. Int. Ed.* **54**, 9654-9658 (2015).
11. Meng, F., Zhong, H., Bao, D., Yan, J., Zhang, X. In situ coupling of strung Co_4N and intertwined N–C fibers toward free-standing bifunctional cathode for bobust, efficient, and flexible Zn–air batteries. *J. Am. Chem. Soc.* **138**, 10226-10231 (2016).
12. Li, G., *et al.* Pomegranate-inspired design of highly active and durable bifunctional electrocatalysts for rechargeable metal-air batteries. *Angew. Chem. Int. Ed.* **55**, 4977-4982 (2016).
13. Li, Y., *et al.* Advanced zinc-air batteries based on high-performance hybrid electrocatalysts. *Nat. Commun.* **4**, 1805 (2013).
14. Tang, C., Wang, B., Wang, H. F., Zhang, Q. Defect engineering toward atomic Co-N_x-C in hierarchical graphene for rechargeable flexible solid Zn-air batteries. *Adv. Mater.* **29**, 1703185 (2017).
15. Xu, N., *et al.* Morphology controlled $La_2O_3/Co_3O_4/MnO_2$–CNTs hybrid nanocomposites with durable bi-functional air electrode in high-performance zinc–air energy storage. *Applied Energy* **175**, 495-504 (2016).
16. Fan, Y., *et al.* Ni-Fe nitride nanoplates on nitrogen-doped graphene as a synergistic catalyst for reversible oxygen evolution reaction and rechargeable Zn-air battery. *Small* **13**, 1700099 (2017).
17. Liu, G., *et al.* An oxygen-vacancy-rich semiconductor-supported bifunctional catalyst for efficient and stable zinc-air batteries. *Adv. Mater.* **31**, 1806761 (2019).
18. Li, Y., *et al.* Atomically thin mesoporous Co_3O_4 layers strongly coupled with N-rGO nanosheets as high-performance bifunctional catalysts for 1D knittable zinc-air batteries. *Adv. Mater.* **30**, 1703657 (2018).
19. Liu, Q., Wang, Y., Dai, L., Yao, J. Scalable fabrication of nanoporous carbon fiber films as bifunctional catalytic electrodes for flexible Zn-Air batteries. *Adv. Mater.* **28**, 3000-3006 (2016).
